# Organizational culture and climate for patient safety in dialysis services: a scoping review

**DOI:** 10.1590/1980-220X-REEUSP-2024-0319en

**Published:** 2025-10-20

**Authors:** Marília Alves Hoffmann, Aline Carrilho Menezes, Tatiana Aparecida Rodrigues, Larissa Assis Caputo Figueiredo, Aldenora Laísa Paiva de Carvalho Cordeiro, Helen Cristiny Teodoro Couto Ribeiro, Luciana Regina Ferreira da Mata

**Affiliations:** 1 Universidade Federal de Minas Gerais, Escola de Enfermagem, Programa de Pós-Graduação em Enfermagem, Belo Horizonte, MG, Brazil.; 2 Universidade Federal de São João Del-Rei, Divinópolis, MG, Brazil.; 3 Universidade Federal de São João Del-Rei, Programa de Pós-Graduação em Enfermagem, Divinópolis, MG, Brazil.; 4 Universidade Federal de Minas Gerais, Escola de Enfermagem, Departamento de Enfermagem Básica, Belo Horizonte, MG, Brazil.

**Keywords:** Organizational Culture, Patient Safety, Renal Dialysis, Patient Care Team

## Abstract

**Objective:**

To map the scientific literature on the assessment of the organizational culture and climate for patient safety in renal dialysis services from the perspective of the multidisciplinary team.

**Method:**

Scope review according to JBI methodology. The articles were extracted from LILACS via VHL, Medline via PubMed, Cochrane Library, Scopus, Web of Science, Embase and gray literature (Google Scholar, CAPES, and university repositories). All descriptors were controlled terms extracted from DeCS/MeSH or Emtree. The guiding question was: which studies available in the literature assessed the organizational culture and climate for patient safety in renal dialysis services from the perspective of the multidisciplinary team?

**Results:**

A total of 13,703 studies were identified, 312 were read in full, and 12 were included. The greatest strengths were in the domains: teamwork, organizational learning and continuous improvement, feedback, and error communication. Weaknesses and opportunities for improvement involved staffing, shift change, leadership support, adverse event reporting, and non-punitive responses to errors. The professional category and type of management influenced the perception of the patient care team.

**Conclusion:**

The findings highlighted areas of weakness in the organizational culture and climate for patient safety in nephrology practices, but did not detail the causal factors, which calls for further studies.

## INTRODUCTION

Hemodialysis is the main renal replacement therapy used in the world, with a prevalence of 89%^(1)^. In Brazil, costs increase with the progression of chronic kidney disease (CKD) and the Brazilian Public Health System’s approach focuses more on dialysis therapies, to the detriment of preventive measures^(2)^. In 2022, Brazil registered approximately 153,831 patients on dialysis therapy, 95.3% on hemodialysis, and 4.7% on peritoneal dialysis, served by 872 active chronic dialysis centers, of which 243 participated in the Brazilian Dialysis Census, with 75.7% being private institutions, 15.6% philanthropic, and 8.6% public^(3)^.

Renal replacement therapy, whether by hemodialysis or peritoneal dialysis, plays an essential role in the management of CKD. However, the increased need for dialysis treatment and the characteristics of CKD make patients more vulnerable to adverse events (AEs) that can compromise health and quality of life^(4)^. Infectious processes, arteriovenous fistula displacement, intradialytic hypotension, arrhythmias, and gas embolism are the most frequent AEs in hemodialysis^(5)^, which contribute to the increase in the mortality rate^(6)^. Conversely, peritoneal dialysis can result in infectious and mechanical complications, such as peritonitis and catheter migration or obstruction, which have the potential to negatively impact treatment safety and efficacy^(7)^. In 2019, 3.16 million people died from CKD in the world^(8)^. In Brazil, between 2009 and 2020, there was an increasing trend in the mortality rate from CKD for both sexes, with an emphasis on the age group over 75 years, mainly in the North and Northeast regions of the country^(9)^.

From this perspective, healthcare provided in dialysis services must be based on the principles of patient safety (PS). PS is structured by activities aiming to promote strategies that direct the organizational culture of health services and the reduction of avoidable risks and harms^(10)^. Established in 2013, the National PS Program aims to ensure the quality of care in all health services in Brazil^(11)^, including dialysis services. Furthermore, as a legislative framework, the following are highlighted: the National Policy for Care for Patients with Kidney Disease^(12)^, the lines of care for people with CKD^(13)^ and for adult patients with CKD^(14)^, and the document from the National Health Regulatory Agency, which establishes the PS culture, risk management, and encouragement of good practices focused on quality and safety in dialysis services^(15)^. This legal basis includes fundamental actions for the prevention, mitigation, and treatment of CKD, with Primary Health Care managing the flow of patients with risk factors and CKD^(12, 13, 14, 15)^.

The multidisciplinary team of dialysis services must develop actions in favor of PS, such as: implementation of guidelines, protocols, and prevention strategies that direct care towards mitigating risks and promoting professionals’ continuing education^(16)^. However, there is a lack of management support and low adherence among healthcare professionals^(17)^. This is a reflection of the organizational culture guiding the workers^(18)^. It is therefore essential to understand the organizational culture and climate focused on PS in dialysis services.

Organizational culture comprises intrinsic values and beliefs, often unnoticed, as they are deep-seated phenomena within organizations. However, it manifests itself through the organizational climate at a given time, being influenced by leadership and both internal and external changes^(19)^. The organizational culture focused on PS is defined as the set of values and behaviors of the professionals who work in the service, being predictive of the quality of care and PS. In turn, the climate of PS consists of the way in which professionals perceive the work environment. Although the concepts present their singularities, it is common to use the terms culture and climate of PS as synonyms in the literature, since they have similar and intersecting outcomes^(20)^. Therefore, it was decided that both terms would be considered in this study.

For a systematic assessment of the perception of the organizational culture and climate for PS, specific instruments are used. In the context of the assessment of the climate of PS, the Safety Attitudes Questionnaire (SAQ)^(21)^ is highlighted. For measuring the PS culture, the following are widely used: Medical Office Survey on Patient Safety Culture (MOSPC)^(22)^ and the Hospital Survey on Patient Safety Culture (HSOPSC)^(23)^, which allow a detailed analysis of practices and perceptions related to PS in health institutions. These instruments play a critical role in collecting reliable data and continuously improving safety strategies in healthcare organizations. They capture professionals’ perceptions of organizational learning from errors, the freedom to report AEs, management’s commitment to PS, and other factors that directly or indirectly impact organizational culture. Furthermore, they enable the identification of weaknesses, strengths, and opportunities for improvement in health services.

The topic is still in its infancy and little discussed in the literature^(24)^, including the specificity of dialysis services. Evaluating the organizational culture and climate for PS in health services from the perspective of the multidisciplinary team contributes positively to the adoption of safe practices within the services, as it allows the construction of an action plan for continuous improvements in the care provided, as well as monitoring of the effects of the interventions carried out^(25,26)^. In this context, mapping and grouping the scientific evidence published on the subject is warranted, and can encourage the development of new studies and strengthen the organizational culture and climate for PS in dialysis services. Thus, the objective of the study was to map the scientific literature on the assessment of the organizational culture and climate for patient safety in renal dialysis services from the perspective of the multidisciplinary team.

## METHOD

### Design Of Study

This is a scoping review conducted according to the JBI methodology^(27)^. The review protocol was registered at Open Science Framework (OSF): https://doi.org/10.17605/OSF.IO/EK93B ^(28)^. To guide writing, the recommendations of the Preferred Reporting Items for Systematic reviews and Meta-Analyses extension for Scoping Reviews (PRISMA-ScR)^(29)^, specific to scope reviews, were followed.

### Guiding Question

The guiding question was “Which studies available in the literature assessed the organizational culture and climate for patient safety in renal dialysis services from the perspective of the multidisciplinary team?” The question was structured based on the acronym PCC - Population, Concept and Context: P-population (multidisciplinary team); C-concept (organizational culture and climate for PS); C-context (dialysis services).

### Selection Criteria

Studies assessing the organizational culture and climate for PS using measurement instruments in dialysis services from the perspective of the multidisciplinary team, available in full, from any geographic area in the databases and gray literature, were included. There was no time limit or language restriction. Review studies, methodological research, comments, institutional reports, event summaries, technical manuals, letters to the editor, case reports, and reviews were excluded. These types of documents did not present primary empirical data or assessment results necessary to identify, describe, or analyze organizational culture and climate. Therefore, they were considered inadequate to answer the research question proposed in this scoping review.

### Data Sources and Search Strategies

The search strategies were outlined with the assistance of a professional librarian. The search was carried out on May 20, 2024, in the databases and institutional repositories Latin American and Caribbean Literature in Health Sciences (LILACS), Nursing Database (BDENF), Spanish Bibliographic Index of Health Sciences (IBECS), and ColecionaSUS via the Virtual Health Library (VHL), Medline via PubMed, Cochrane Library, Scopus, Web of Science and Embase. The databases were accessed through the CAPES Journal Portal, through the Federated Academic Community (CAFe). A search was also carried out in the gray literature Google Scholar, in the Catalog of Theses and Dissertations of the Coordination for the Improvement of Higher Education Personnel (CAPES) and in the University Repositories (UFC, UFAL, UNB, USP and UFMG). These institutional repositories were selected due to the quality and relevance of their content, as well as the significant role of these institutions in the production and dissemination of knowledge, contributing to the advancement of open science and the promotion of innovative research. The search strategies were adapted according to the needs of the databases searched.

For selecting the descriptors, DeCS (Health Sciences Descriptors)/MeSH (Medical Subject Headings) were used, and for Embase, Emtree was used. All descriptors included in the search strategy were controlled terms, that is, extracted from DeCS/MeSH or Emtree. The Boolean operators used were OR and AND. The OR operator was applied to find records containing one of the separate terms (e.g.: *hemodialysis or dialysis*) and AND to find records containing all the specified terms (e.g., *hemodialysis and ‘patient safety*’), as shown in Chart 1.

**Chart 1:** Search terms and strategies applied to each database – Belo Horizonte, MG, Brazil, 2024.

Databases and search date	Search Strategy	Number of studies retrieved
Lilacs (via VHS) May 20, 2024	(“*Renal Dialysis*” OR “*Diálisis Renal*” OR “Diálise Renal” OR “Diálise Extracorpórea” OR “Hemodiálise” OR “*Dialysis*” OR “*Diálisis*” OR “Diálise” OR “*Hemodialysis Units Hospital*” OR “*Unidades de Hemodiálisis en Hospital*” OR “Unidades Hospitalares de Hemodiálise” OR “Unidades Hospitalares de Diálise Renal” AND (“*Patient Safety*” OR “*Seguridad del Paciente*” OR “Segurança do Paciente” OR “*Organizational Culture*” OR “Cultura Organizacional” OR “Cultura Organizacional” OR “Cultura Corporativa” OR “*Safety Management*” OR “*Administración de la Seguridad*” OR “Gestão da Segurança”) AND (db:(“LILACS” OR “IBECS” OR “BDENF” OR “colecionaSUS”) AND la:(“en” OR “es” OR “pt”))	96
MEDLINE via PUBMED May 20, 2024	(“*Renal Dialysis*” OR “*Dialysis*” OR “*Hemodialysis Units Hospital*”) AND (“*Patient Safety*” OR “*Organizational Culture*” OR “*Safety Management*”)	597
*Cochrane Library* May 20, 2024	(“*Renal Dialysis*” OR “*Dialysis*” OR “*Hemodialysis Units Hospital*”) AND (“*Patient Safety*” OR “*Organizational Culture*” OR “*Safety Management*”)	102
*Scopus* May 20, 2024	(“*Renal Dialysis*” OR “*Dialysis*” OR “*Hemodialysis Units Hospital*”) AND (“*Patient Safety*” OR “*Organizational Culture*” OR “*Safety Management*”)	1745
*Web of Science* May 20, 2024	(“*Renal Dialysis*” OR “*Dialysis*” OR “*Hemodialysis Units Hospital*”) AND (“*Patient Safety*” OR “*Organizational Culture*” OR “*Safety Management*”)	389
*Embase* May 20, 2024	*hemodialysis* or *dialysis* AND *‘patient safety’*	2708
**Gray literature and search date**	**Search Strategy**	**Number of studies retrieved**
*Google Scholar* May 20, 2024	(Percepção) AND (“Cultura de segurança do paciente” OR “Clima de segurança do paciente”) AND (“Serviços de diálise”)	8000
CAPES Catalog of Theses & dissertations May 20, 2024	(Percepção) AND (“Cultura de segurança do paciente” OR “Clima de segurança do paciente”) AND (“Serviços de diálise”)	0
Repositories (UFC, UFAL, UNB, USP, UFMG) May 20, 2024	(Percepção) AND (“Cultura de segurança do paciente” OR “Clima de segurança do paciente”) AND (“Serviços de diálise”)	66

### Study Selection

Two researchers independently selected the studies and a third one evaluated the disagreements until final consensus was reached. The references manager Endnote was used, where duplicates were excluded. Then, the list of references was exported to the bibliographic manager Rayyan Qatar Computing Research Institute *(Rayyan* QCRI); the study selection process took place in three stages: analysis of titles, reading of abstracts and reading of the studies in full. These stages were guided by inclusion and exclusion criteria. The same process was used to select articles from the gray literature, with manual searches of the reference lists of the included studies being analyzed as a strategy to identify scientific productions that had not been previously retrieved.

### Data Extraction and Analysis

A database was created by researchers using the software *Microsoft Excel®* 2016 version, for extraction of the following data: author/year of publication; title; country where the study was conducted; objectives; sample; participant adherence rate; population analyzed; study location; instrument used to assess the organizational culture or climate for PS; level of culture or climate of PS; strengths, weaknesses, and opportunities for improvement for organizational culture and climate for PS in dialysis services.

The extracted data and the main characteristics of the selected articles were compiled in Chart 2. To analyze and categorize the results of studies that used the HSOPSC^(23)^ and the MOSPC^(22)^ and their adapted versions, the evaluation criteria proposed by the Agency for Healthcare Research and Quality (AHRQ)^(23)^ were used: areas considered strong or strengths are those that have 75% or more positive response percentages (PRP) from the multidisciplinary team, and areas considered fragile or with weaknesses are those with PRPs equal to or less than 50%. Areas evaluated with <75% and >50% are considered as opportunities for improvements in organizational culture and climate for PS^(23)^. For the analysis of the adapted version of the SAQ instruments^(30)^ and Safety Climate Survey (SCS)^(31)^, the authors considered strong areas those with 75% or more of PRP and fragile areas or areas with weaknesses those with PRP of less than 75%^(30,31)^.

**Chart 2:** Characterization of studies included in the scoping review (n = 12) – Belo Horizonte, MG, Brazil, 2024.

Author, year and country	Sample and adherence rate	Objectives of the studies	Population	Service location
Hoffmann et al.^(32)^ (2023); Brazil	n = 56 (%) = 71.8	To assess the culture of PS from the perspective of the nursing team in dialysis services during the COVID-19 pandemic.	Nurses, nursing technicians and assistants.	Three dialysis services in Minas Gerais
Rodrigues et al.^(24)^ (2023); Brazil	n = 134 (%) = 86	To evaluate the factors associated with the culture of PS in dialysis during the COVID-19 pandemic.	Nurses, nursing technicians and assistants, social workers, nutritionists, psychologists, pharmacists, and the administrative sector.	Three dialysis services in Minas Gerais
Silva^(33)^ (2019); Brazil	n = 32 (%) = 89	To evaluate the PS culture of professionals in a hemodialysis service at a teaching hospital.	Nurses, nursing technicians and assistants, doctors, administrative staff, nutritionists, cleaning and sanitation staff, psychologists, social workers, receptionists, and water technicians.	University Hospital of Bahia
Millson; Hackbarth; Benard^(34)^ (2019); USA	n = 99 (%) ≥80	To assess hemodialysis staff perceptions of PS culture, implement the AHRQ safety program, and promote increased staff adherence to infection prevention standards.	Nurses and nursing assistants and technicians	Six New York City outpatient dialysis clinics
Grilo^(35)^ (2018); Portugal	n = 108 (%) = 73	To analyze healthcare professionals’ perceptions of the culture of PS; compare perceptions among different professional categories; and identify areas for improvement of the culture of PS.	Nurses, nursing assistants and technicians, doctors and administrators	Hemodialysis clinic in the Lisbon region
Izquierdo et al.^(36)^ (2018); Spain	n = 111 (%) = 85	To determine the frequency of perceptions and attitudes of professionals in hemodialysis centers regarding PS; and to identify strengths and opportunities for improvement for PS.	Nurses, nursing assistants and other categories	Six hemodialysis centers in Castile-La Mancha
Dantas^(37)^ (2018); Brazil	n = 38 (%) = 100	To evaluate the PS culture in hemodialysis units from the perspective of the nursing team; and to quantify the events reported by this team.	Nurses, nursing assistants and technicians	Two hemodialysis hospital units in Ceará
Davis et al.^(38)^ (2016); USA	n = 581 (%) = 97	To assess the perceptions of the multidisciplinary team regarding the culture of PS in hemodialysis clinics.	Administrative sector, doctors, nutritionist, social worker, nurses and nursing technicians	Hemodialysis clinics in two US cities
Ulrich; Kear^(39)^ (2015); USA	n = 929 (%) = 95	To analyze the perceptions of nephrology nurses about PS and its culture, considering the work unit, the organizational environment and the role played.	Nurses	American Association of Nephrology Nurses
Ulrich; Kear^(40)^ (2014); USA	n = 929 (%) = 95	To describe the results of PS culture in nephrology nursing practice settings; and to compare the PS culture data obtained with data published by AHRQ.	Nurses	American Association of Nephrology Nurses
Taher et al.^(41)^ (2014); Saudi Arabia	n = 509 (%) = 65	To assess the PS climate perceived by nurses and physicians in dialysis units in Saudi Arabia.	Nurses and doctors	Five dialysis units in three cities in Saudi Arabia
Di Benedetto et al.^(42)^ (2011); Italy	n = 346 (%) = 89	To assess the level of PS climate in the dialysis clinic network and the healthcare team’s adherence to the implementation of standard procedures to improve safety in the dialysis service.	Nurses, care assistants and physicians	Thirty-three dialysis centers of the *Fresenius Medical Care* in Italy

PS: Patient Safety; COVID-19: Coronavirus Disease 2019; AHRQ: Agency for Healthcare Research and Quality; USA: United States of America.

The selected studies were organized by year of publication, and the areas of strengths, opportunities for improvement, and weaknesses were presented together with the measurement instrument used in chart format.

All researchers participated in the interpretation and synthesis of the articles data, using narrative discussion to associate the tabulated results with the objective and guiding research question.

## RESULTS

**Figure 1:**
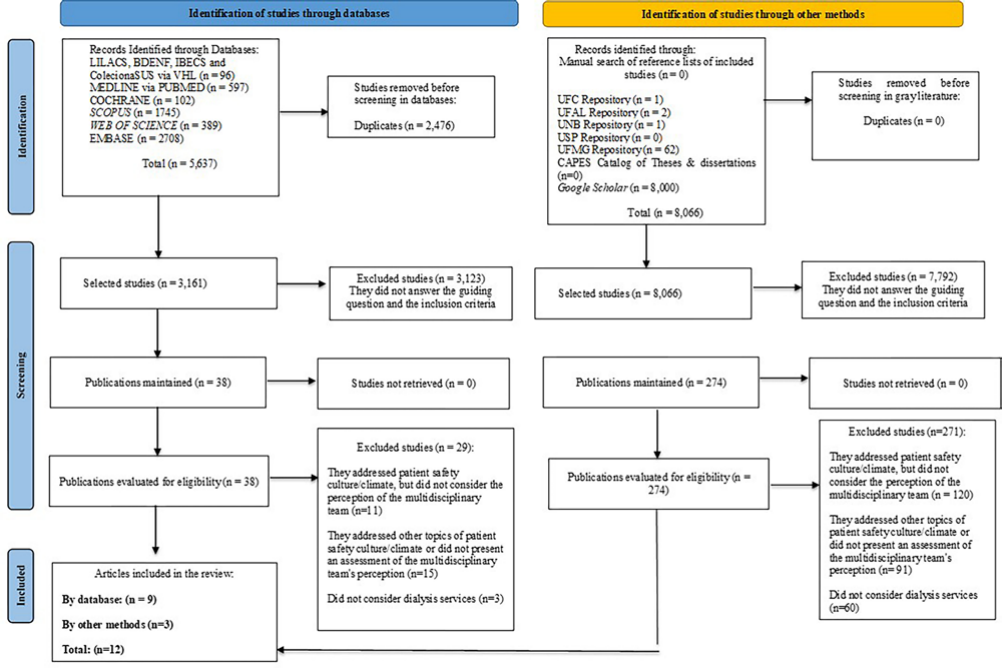
PRISMA-ScR flowchart for selecting studies for inclusion in the scoping review – Belo Horizonte, MG, Brazil, 2024.

A total of 5,637 studies were found in the searched databases. After removing 2,476 duplicates, 3,161 studies remained. Of these, 3,123 studies were excluded after reading of the titles and abstracts No evidence was selected after manual searching of the reference list of included studies. Of the 38 studies selected for full reading, 29 were excluded with justifications, which resulted in the inclusion of nine studies. In the gray literature, 8,066 studies were identified. After reading the titles and abstracts, 7,792 studies were excluded because they did not answer the guiding question and did not meet the inclusion criteria of this scoping review. Therefore, 274 were assessed for eligibility, of which 271 were excluded based on justifications, which resulted in the inclusion of three studies in the sample. Finally, 13,703 studies were found, 312 were read in full and 12 were selected to compose the final sample. This entire process was described in the PRISMA ScR flowchart (Figure 1).

The 12 studies^(24,32, 33, 34, 35, 36, 37, 38, 39, 40, 41, 42)^ included in this scoping review presented a quantitative methodological approach and cross-sectional design, whose objectives were detailed in Chart 2. The publication period was between 2011 and 2023 and all were developed in public, private, or philanthropic dialysis services. Most of the research was of North American and Brazilian origin (four studies each, corresponding to 33.4% of the total in both cases). The adherence rate of participants to the studies varied between 65% and 100%, with an average of 85.5%. Within the multidisciplinary team, the composition of participants differed between the studies analyzed; two American studies^(39–40)^ exclusively evaluated the perception of nephrology nurses (Chart 2).

Regarding the assessment instruments, the majority (n = 9; 75%) used instruments to assess the organizational culture for PS, among which the HSOPSC^(23)^ was the most referenced^(24,32, 33, 34, 35, 36,38, 39, 40)^. The others (n = 3; 25%) used instruments to measure the organizational climate for PS, with emphasis on the SAQ^(37)^ and the SCS^(41,42)^.

The main strengths identified were related to teamwork in dialysis services^(35,36,38, 39, 40)^. Following this, organizational learning and continuous improvement (training) stood out, as well as feedback and communication of errors, present in 75% of the studies (n = 9)^(24, 25, 26, 27, 28, 29, 30, 31, 32, 33, 34, 35, 36,38, 39, 40, 41)^. Three international studies (25%)^(38, 39, 40)^ highlighted the importance of actions for infection control in nephrology practices as areas strengthened for organizational culture and climate for PS in dialysis services (Chart 3).

**Chart 3:** Dimensions of organizational culture and climate for patient safety (n = 12) – Belo Horizonte, MG, Brazil, 2024.

Articles	Instrument of assessment of organizational culture and climate for PS	Categories	Dimensions of instruments for assessing organizational culture and climate for PS
Hoffman et al.^(32)^ (2023); Brazil	Adapted HSOPSC	Strengths	Expectations and actions to promote safety of management/supervision/unit managers/service (78%)
		Opportunities for improvement	Organizational learning/continuous improvement (69%) Feedback and error communication (63%) Teamwork within units/service (63%) Openness of communication (55%)
		Weaknesses	Internal transfers and shift changes (49%) Staffing (45%) General perception of PS (45%) Hospital management support for PS (48%) Teamwork within units (45%) Frequency of reported adverse events (43%) Non-punitive responses to errors (22%)
Rodrigues et al.^(24)^ (2023); Brazil	Adapted HSOPSC	Strengths	Organizational learning/continuous improvement (86%) Expectations and actions to promote safety of management/supervision/unit managers/service (77%)
		Opportunities for improvement	Teamwork within units/service (73%) Hospital management support for PS (64%) Feedback and error communication (60%) Frequency of reported adverse events (59%) Internal transfers and shift changes (57%) Openness of communication (56%) Teamwork within units (56%) General perception of PS (54%)
		Weaknesses	Staffing (49%) Non-punitive responses to errors (29%)
Silva^(33)^ (2019); Brazil	Adapted HSOPSC	Strengths	Organizational learning and continuous improvement (84%)
		Opportunities for improvement	Teamwork within units/service (74%) Staffing (58%)
		Weaknesses	Non-punitive responses to errors (47%) *Feedback* and error communication (46%) Hospital management support for PS (45%) Frequency of reported adverse events (44%) Openness of communication (38%) Management safety promotion expectations and actions/supervision/unit managers/service (35%) Teamwork within units (32%) General perception of PS (26%) Internal transfers and shift changes (19%)
Millson; Hackbart; Benard (2019)^(34)^; USA	Adapted HSOPSC	Strengths	Organizational learning and continuous improvement (80%) Openness of communication (77%)
		Opportunities for improvement	Hospital management support for PS (63%) Teamwork within units (57%)
		Weaknesses	Not mentioned
Grilo^(35)^ (2018); Portugal	Adapted HSOPSC	Strengths	Teamwork within units (81%) Organizational learning and continuous improvement (77%) Hospital management support for PS (75%) Teamwork within units (75%)
		Opportunities for improvement	General perception of PS (72%) Expectations and actions to promote safety of management/supervision/unit managers/service (67%) Internal transfers and shift changes (61%) Staffing (55%) Frequency of reported adverse events (58%)
		Weaknesses	Feedback and error communication (50%) Openness of communication (50%) Non-punitive responses to errors (37%)
Izquierdo et al.^(36)^ (2018); Spain	Adapted HSOPSC	Strengths	Teamwork within units (86%) *Feedback* and error communication (76%) Expectations and actions to promote safety of management/supervision/unit managers/service (75%)
		Opportunities for improvement	Frequency of reported adverse events (73%) Organizational learning and continuous improvement (71%) General perception of PS (70%) Openness of communication (65%) Staffing (63%) Non-punitive responses to errors (63%) Internal transfers and shift changes (62%) Hospital management support for PS (59%) Teamwork within units (55%)
		Weaknesses	Not mentioned
Dantas^(37)^ (2018); Brazil	Adapted SAQ	Strengths	Job satisfaction (86%) Teamwork climate (82%) Working conditions (79%)
		Opportunities for improvement	Not mentioned
		Weaknesses	Climate of PS (72%) Perception of stress (67%)
Davis et al.^(38)^ (2016); USA	Adapted HSOPSC	Strengths	Hospital management support for PS (88%) When there is a lot to be done quickly, we can work as a team (86%) Managers consider workers’ suggestions on measures to prevent infections associated with vascular access (84%) Ways to avoid repeating errors in the unit are discussed (83%) We are actively modifying protocols and policies to reduce vascular access-related infections at our institution (82%) After we made changes to improve PS, we evaluated its effectiveness (81%) My supervisor or manager provides positive feedback when he identifies that the work was carried out in accordance with the procedures established for PS (79%) Workers feel comfortable speaking up if they notice anything that could increase the risk of vascular access-related infections (76%) Errors can lead to positive change (76%) Treat each other with respect (75%)
		Opportunities for improvement	Not mentioned
		Weaknesses	Not mentioned
Ulrich; Kear^(39)^ (2015); USA	Adapted HSOPSC and MOSPC	Strengths	Teamwork (87%) Team training (80%) Infection control in dialysis (76%) Continuous leadership - organizational learning (76%)
		Opportunities for improvement	Hospital management support for PS (74%) Safety promotion expectations and actions of management/supervision/unit managers/service (70%) Process standardization (69%) General perception of PS (65%) Openness of communication (64%) Feedback and error communication (63%) Organizational leadership support for PS (60%) Frequency of reported adverse events (55%) Non-punitive responses to errors (55%) Staffing (54%)
		Weaknesses	Not mentioned
Ulrich; Kear^(40)^ (2014); USA	Adapted HSOPSC	Strengths	Teamwork between units (81%) Infection control (79%) Team training (78%)
		Opportunities for improvement	Organizational learning and continuous improvement (73%) Expectations and actions to promote safety of management/supervision/unit managers/service (72%) Hospital management support for PS (67%) General perception of PS (65%) Process standardization (62%) Openness of communication (60%) Feedback and error communication (59%) Staffing (57%) Organizational support from leadership for PS (56%) Non-punitive responses to errors (55%) Frequency of reported adverse events (55%)
		Weaknesses	Internal transfers and shift changes (33%)
Taher et al.^(41)^ (2014); Saudi Arabia	SCS	Strengths	Passing on information at the beginning of the shift is important for PS (85%) Staff takes responsibility for PS (84%) PS is considered a priority (83%) Communication of information is frequent in this environment (81%) I am familiar with the channels for asking questions about PS issues (79%) Adverse events are often associated with multiple system failures (78%) The physicians and nurse leaders in my clinical area listen to me and consider my concerns (77%) I feel encouraged by my colleagues to report any concerns I may have about PS (76%) More efforts for PS were made compared to last year (76%)
		Opportunities for improvement	Not mentioned
		Weaknesses	Leadership is directing us to act as a PS-centered institution (74%) Medical errors are adequately treated (69%) The PS culture of this clinical area encourages learning based on errors (68%) I would feel safe being treated at this facility (67%) I get appropriate feedback about my performance (64%) Management does not ignore PS issues in favor of productivity (62%) My suggestions about PS are considered by the management (68%)
Di Benedetto et al.^(42)^ (2011) – Italy	SCS	Strengths	Leadership is directing us to act as a PS-centered institution (93%) The physicians and nurse leaders in my clinical area listen to me and consider my concerns (83%) I am encouraged by my colleagues to report any concerns I may have about PS (85%) The PS culture of this clinical area encourages learning based on errors (82%)
		Opportunities for improvement	Not mentioned
		Weaknesses	Not mentioned

PS: Patient Safety; COVID-19: Coronavirus Disease 2019; AHRQ: Agency for Healthcare Research and Quality; USA: United States of America.

The type of service management was also evaluated as a factor that influences the professionals’ perception. Two Brazilian studies (17%)^(24,32)^ found the work factors predisposing to the organizational culture and climate for PS in nephrological care practices. One of the studies^(24)^ identified more strengths in privately and philanthropically managed dialysis services; weak areas, in turn, were more frequently reported in the public management service, according to the perception of the multidisciplinary team. However, another study^(32)^ pointed out that the nursing team of the public dialysis service presented a better perception of the organizational culture and climate for PS when compared to the teams of philanthropic and private dialysis services.

In most studies (n = 8; 67%), the general perception of the organizational culture and climate for PS was assessed by participants as positive^(24,32,35,38, 39, 40, 41, 42)^. Nevertheless, two Brazilian studies (n = 2; 17%) assessed the general perception as predominantly negative^(33.37)^.

## DISCUSSION

Based on the results, it was identified that the most recent studies on the assessment of organizational culture and climate for PS in dialysis services were published between 2019 and 2023, of which three were Brazilian^(24,32,33)^ and one was American^(34)^. It should be noted that two studies were developed during the *Coronavirus Disease* 2019 (COVID-19) pandemic in Brazil, one focusing on the perception of the nursing team^(32)^ and the other in the multidisciplinary team^(24)^. Thus, given the numerous difficulties experienced in the pandemic context for scientific production in this area, it is essential that managers and public policy makers promote the construction and implementation of strategies to improve the organizational culture and climate for PS in nephrology practice environments^(16)^.

Most studies were conducted in developed countries, where organizational culture and climate for PS were better evaluated compared to studies conducted in low- or middle-income countries^(38,42)^. Brazil was the only country in Latin America where it was possible to find studies on the subject. Given this scenario, a discussion arises about the negative impact of socioeconomic and geographic inequalities on the perception of organizational culture and climate for PS by the multidisciplinary team of dialysis services. Similarly, studies have shown that various inequalities negatively impact access to health services and the quality of care provided, constituting a public health problem^(43,44)^.

Assessing the organizational culture and climate for PS should be a priority for managers and professionals in health services, as this allows the identification and measurement of organizational conditions that can lead to an increase in AEs and impact the quality of care provided, besides stimulating the development of strategies to improve PS in the Health Care Network^(45)^. Instruments for assessing the culture and climate of PS can guide the understanding and transformation of attitudes and behavior related to PS, based on the perception of professionals in a healthcare organization^(45)^.

The areas of greatest strength for the organizational culture and climate for PS, which stood out, were: organizational learning and continuous improvement; feedback and communication about errors; and teamwork. A positive organizational culture and climate for PS can direct team behavior toward providing safe care. Therefore, the areas of strength highlighted in the studies should be priorities in health services. Thus, it contributes to encouraging professionals to carry out error and AE analysis, with the aim of continuously improving PS in health services^(46)^.

Specifically in dialysis services, professionals recognize that strengthening the organizational culture and climate for PS allows for the improvement and development of essential skills for safe care practice^(24)^. In this respect, health services must replace the punitive culture of error with a culture of learning, which encourages open communication of incidents without repression or punishment^(47)^.

The power of teamwork connects the knowledge and skills of different people with a single purpose: patient care^(45)^. However, the work of the multidisciplinary team in health presents challenges such as the existence of conflicts, individualism, and the hierarchization of relationships^(48)^. It is important that professionals adopt measures for the adequate development of teamwork, with a focus on contributing to the quality and access to health services^(49)^.

International studies^(38, 39, 40)^ demonstrated the prevention of infections in dialysis services to promote the improvement of the organizational culture and climate for PS. Thus, the risk of infection is considered one of the main causes of mortality and hospitalization in patients undergoing dialysis treatment^(5)^. Strategies such as the implementation of investigative (root cause) analysis to determine system and process problems are necessary to reduce and mitigate risks and AEs in dialysis services^(16)^. Furthermore, specific actions in the area of hand hygiene and care of central venous catheters must be prioritized for effective infection control^(16,26)^.

Brazilian studies^(24,32,33,37)^ presented areas related to the general perception of organizational culture and climate for PS as a weakness or opportunity for improvement. This may be associated with the professional challenges of Brazilian public services, such as the high demand for services and the lack of greater investment in human and technological resources^(50)^.

Three studies (25%) highlighted staffing and shift transition/transfer/handover within dialysis units and services as weaknesses^(24,32,40)^. However, four studies (33%) identified these areas as opportunities for improvement in organizational culture and climate for PS^(33,35,36,39)^. In two studies (17%), working conditions were considered strong areas^(37,41)^. It should be noted that the staffing of dialysis services in Brazil is provided for by the Collegiate Board Resolution (RDC) No. 11/2014, which also establishes requirements for good operating practices for dialysis services^(51)^.

A Brazilian study with the nursing team showed that inadequate working conditions, resulting from the environment and the functions performed, negatively impact the perception of the organizational culture and climate for PS^(52)^. The work environment generates physical and mental exhaustion due to the lack of professional recognition, lack of motivation, lack of inputs, inadequate remuneration, excessive workload, incorrect staffing, night shifts, and employee dissatisfaction^(52)^.

It is important to note that the handover is the moment when the most important information related to the patient’s progress is discussed by the multidisciplinary team, which can contribute to the continuity of care and the guarantee of PS, as long as it is developed through effective communication^(53)^. To this end, standardization through protocols and communication tools is essential to ensure the continuity of actions, in addition to the encouragement of changes in attitudes, and strengthening of the organizational culture and climate^(52)^.

The support of managers and leadership for the concerns, demands, and suggestions of the multidisciplinary team working in dialysis services was highlighted as a weak dimension or as an opportunity for improvement in 75% (n = 9) of the studies^(24,32, 33, 34,36,39, 40, 41, 42)^. The participation of leadership in accepting the demands and suggestions of the multidisciplinary team is extremely important in promoting PS. An Iranian study conducted in hospitals indicates that managers, leaders, and supervisors should promote actions favoring the development of values, beliefs, and personal behavior aligned with the values of the organizational culture and climate. The leader’s commitment to identifying problems in the work process and developing strategies that support resources and learning opportunities can contribute to improving the provision of health care^(46)^.

Additionally, the management model adopted by the healthcare organization can influence professionals’ perceptions of the organizational culture and climate for PS. In this respect, everyone must be committed to maintaining and promoting the culture and climate of PS. Hence, management plays an essential role in improving the organizational culture and climate for PS, as it promotes the organization of flows that improve the effectiveness of health work^(54)^.

The dimensions of organizational culture and climate for PS “frequency of reported AEs” and “non-punitive responses to errors” were cited as opportunities for improvement or weaknesses in seven studies (58%)^(24,32,33,35,36,39,40)^. The dimension “AE are often associated with multiple system failures” was considered a strength in three (25%) surveys^(36,38,41)^. It is therefore evident that the existence of a punitive culture and work overload inhibit professionals from reporting AEs. These results are in line with another study, in which punitive culture and excessive workload are also considered obstacles to AE reporting^(53)^. In these circumstances, underreporting of AEs should be addressed as an opportunity for improvement, as it impedes the development of actions to prevent the recurrence of healthcare errors and the diagnosis of service risks.

The influence of the professional category on the perception of organizational culture and climate for PS was analyzed by studies with different results. In five articles (42%), administrators/managers/leaders presented a more positive overall perception of the organizational culture and climate for PS when compared to the rest of the multidisciplinary dialysis team^(24,35,38,40,42)^. Two studies (17%) found that physicians, multidisciplinary teams, and administrative staff reported more areas of strength when compared to the nursing team^(24,38)^. Two Brazilian studies highlighted that nurses, when compared with doctors or nursing assistants and technicians, presented a better assessment of the perception of organizational culture and climate for PS^(32,37)^. A study conducted in Saudi Arabia also corroborates this finding^(41)^. In contrast, a Brazilian study^(33)^ points out that the perception among nursing assistants and technicians was better evaluated compared to that of nurses and doctors in dialysis services. Two American studies evaluated the organizational culture and climate for PS from the perspective of 929 nurses working in dialysis services^(39,40)^. Nurses’ perception is considered essential, given their proximity to patients, leadership role, and central role in infection control and effective communication within the team^(24,39,40)^.

Therefore, the influence of the professional category on the perception of culture and organizational climate for PS in dialysis services was noted in the studies. However, it was not possible to identify a pattern of how these influences occur from the studies mapped. An international study found that managers and administrative staff had a more positive perception of the culture and climate of PS when compared to other members of the multidisciplinary team^(55)^.

In this regard, variability was observed in the perception of the multidisciplinary team in the Brazilian scenario. A study found a more positive perception of the organizational culture and climate for PS in dialysis services by managers and the administrative sector^(24)^. Two studies highlighted the perception of nurses compared to physicians and other members of the nursing team^(32,37)^. Finally, one identified a better perception among nursing technicians and assistants compared to nurses and doctors^(33)^. However, inequalities in professionals’ perception of the organizational culture and climate for PS need to be corrected through their professional appreciation and discussions about practices that encourage safe care^(56)^.

Furthermore, the topic has to be addressed in the national curricular guidelines and in the political-pedagogical plan of professional training in the health area during the teaching- learning process, as it is essential for strengthening the culture and climate of PS, given that current students will be the future professionals in health services^(57)^, including dialysis services.

The professional category also influenced the perception of organizational culture and climate for PS according to the management model of dialysis services^(24,32)^. Such differences can be justified by some hypotheses, since private institutions receive greater investment from managers in actions to improve work processes and the quality of care provided due to market competitiveness^(24)^. In contrast, the nursing team develops skills and competencies aimed at identifying risks involving PS due to the proximity to the patient during the care provided, when compared to other professional categories^(52)^. Finally, the public dialysis service during the pandemic had training on care flows because it is a reference hospital for COVID-19 treatment, which may have influenced the perception of the nursing team^(32)^.

Therefore, the outcomes of the mapped studies showed that the organizational culture and climate for PS in dialysis services are perceived by the multidisciplinary team as relatively positive or neutral (opportunities for improvement). However, a positive organizational culture and climate for PS is expected across all services, as their consolidation is essential to improving the quality of care. To achieve this, managers and leaders must recognize the importance of communication to identify the concerns of both healthcare professionals and patients^(52)^. In addition, the collaboration of the multidisciplinary team is essential to maintain strengths, enhance opportunities for improvement, and repair weaknesses in the areas of organizational culture and climate for PS in dialysis services.

It was noted that the literature on this topic is incipient and needs to be valued in the global context, as few studies were identified that evaluated the organizational culture and climate of PS in dialysis services from the perception of the multidisciplinary team. A limitation is the fact that two studies included^(39,40)^ exclusively addressed the perception of nurses, which may restrict the generalization of the findings to the entire multidisciplinary team.

Another important point is the lack of consensus in the literature on the use of the terms organizational “culture” and “climate” for PS, an aspect that hindered the data analysis process of the studies in this review. The researchers minimized the difficulty by considering both terms, since they are intrinsically correlated and reflect on the care and management practice of dialysis services. However, this review will contribute to the clinical and managerial practice of dialysis services as it promotes increased understanding of the organizational culture and climate for PS from the perspective of the multidisciplinary team.

## CONCLUSION

The areas of greatest strength for the organizational culture and climate for PS were in the areas of teamwork, organizational learning and continuous improvement, feedback and communication of errors. Weaknesses and opportunities for improvement involved staffing, shift change and internal transferences, leadership support, adverse event reporting, and non-punitive responses to errors.

The professional category, geographic location, and type of management of dialysis services influenced the perception of organizational culture and climate for PS, and these were positive in most studies. The findings of this review may assist managers, leaders, and professionals in strategically planning the care provided in dialysis services, based on the assessment of the organizational culture and climate for PS, with the aim of proposing actions for continuous improvement of PS and health care. Thus, it will contribute to directing future studies that promote the strengthening of the culture and climate of PS and safe nephrological practices within the scope of Brazilian public health. The findings pointed to areas of weakness, but did not present a detailed understanding of the factors that contribute to such results, which raises the need for further studies to obtain a better characterization of how the multidisciplinary team evaluates and perceives the organizational culture and climate for PS in renal dialysis services, as well as a possible generalization of the results.

## DATA AVAILABILITY

The entire data set supporting the results of this study was made available and published in the article itself.
