# Supplementary material for: Organizational culture and climate for patient safety in dialysis services: a scoping review
Source: Rev Esc Enferm USP. 2025 Oct 20;59:e20240319. doi: 10.1590/1980-220X-REEUSP-2024-0319en (PMC12543328; doi:10.1590/1980-220X-REEUSP-2024-0319en)
Supplement: Translation

# Translation: Cultura e clima organizacional para segurança do paciente em serviços de diálise: revisão de escopo

**DOI:** 10.1590/1980-220X-REEUSP-2024-0319pt

**Authors:** Marília Alves Hoffmann, Aline Carrilho Menezes, Tatiana Aparecida Rodrigues, Larissa Assis Caputo Figueiredo, Aldenora Laísa Paiva de Carvalho Cordeiro, Helen Cristiny Teodoro Couto Ribeiro, Luciana Regina Ferreira da Mata

**Affiliations:** 1 Universidade Federal de Minas Gerais, Escola de Enfermagem, Programa de Pós-Graduação em Enfermagem, Belo Horizonte, MG, Brasil.; 2 Universidade Federal de São João Del-Rei, Divinópolis, MG, Brasil.; 3 Universidade Federal de São João Del-Rei, Programa de Pós-Graduação em Enfermagem, Divinópolis, MG, Brasil.; 4 Universidade Federal de Minas Gerais, Escola de Enfermagem, Departamento de Enfermagem Básica, Belo Horizonte, MG, Brasil.

**Keywords:** Cultura Organizacional, Segurança do Paciente, Diálise Renal, Equipe de Assistência ao Paciente

## Abstract

**Objetivo:**

Mapear a literatura científica sobre avaliação da cultura e do clima organizacional para segurança do paciente em serviços de diálise renal na perspectiva da equipe multidisciplinar.

**Método:**

Revisão de escopo conforme metodologia JBI. Os artigos foram extraídos do LILACS via BVS, *Medline* via PubMed, *Cochrane Library*, *Scopus*, *Web of Science*, *Embase* e literatura cinzenta (*Google Scholar*, CAPES e repositórios de universidades). Todos os descritores foram termos controlados extraídos do DeCS/MeSH ou Emtree. A questão norteadora foi: quais estudos disponíveis na literatura avaliaram a cultura e o clima organizacional para segurança do paciente em serviços de diálise na perspectiva da equipe multidisciplinar?

**Resultados:**

Foram identificados 13.703 estudos, 312 foram lidos na íntegra e 12 incluídos. As maiores fortalezas foram nos domínios: trabalho em equipe, aprendizado organizacional e melhoria contínua, *feedback* e comunicação sobre erro. As fragilidades e as oportunidades de melhoria envolveram dimensionamento, transição de plantão, apoio da liderança, notificação de eventos adversos e respostas não punitivas aos erros. A categoria profissional e o tipo de gestão influenciaram na percepção da equipe de assistência ao paciente.

**Conclusão:**

Os achados apontaram áreas de fragilidades para cultura e clima organizacional para segurança do paciente nas práticas nefrológicas, mas não detalharam os fatores causais, o que suscita a condução de novos estudos.

## INTRODUÇÃO

A hemodiálise é a principal terapia substitutiva renal utilizada no mundo, com prevalência de 89%^(1)^. No Brasil, os custos aumentam com a progressão da doença renal crônica (DRC) e a abordagem do Sistema Único de Saúde concentra-se mais nas terapias dialíticas, em detrimento das medidas preventivas^(2)^. Em 2022, o Brasil registrou aproximadamente 153.831 pacientes em terapia dialítica, sendo 95,3% em hemodiálise e 4,7% em diálise peritoneal, atendidos por 872 centros ativos de diálise crônica, dos quais 243 participaram do Censo Brasileiro de Diálise, com 75,7% de natureza privada, 15,6% filantrópica e 8,6% pública^(3)^.

A terapia renal substitutiva, seja por hemodiálise ou diálise peritoneal, desempenha um papel essencial no manejo da DRC. Contudo, o aumento da necessidade de tratamento dialítico e as características da DRC tornam os pacientes mais vulneráveis a eventos adversos (EA) que podem comprometer a saúde e a qualidade de vida^(4)^. Processos infecciosos, deslocamento da fístula arteriovenosa, hipotensão intradialítica, arritmias e embolia gasosa são os EA mais frequentes na hemodiálise^(5)^, o que contribui para o aumento da taxa de mortalidade^(6)^. Em contrapartida, a diálise peritoneal pode resultar em complicações infecciosas e mecânicas, como peritonite e migração ou obstrução do cateter, as quais têm o potencial de impactar negativamente a segurança e a eficácia do tratamento^(7)^. Em 2019, 3,16 milhões de pessoas morreram devido à DRC no mundo^(8)^. No Brasil, entre os anos 2009 a 2020, houve uma tendência crescente da taxa de mortalidade por DRC para ambos os sexos, com ênfase na faixa etária acima de 75 anos, principalmente nas regiões do Norte e Nordeste do país^(9)^.

Sob essa perspectiva, a assistência de saúde prestada nos serviços de diálise deve estar baseada nos princípios da segurança do paciente (SP). A SP é estruturada por atividades que visam à promoção de estratégias que direcionem a cultura organizacional dos serviços de saúde e a redução dos riscos e danos evitáveis^(10)^. Instituído em 2013, o Programa Nacional de SP objetiva garantir a qualidade do cuidado em todos os serviços de saúde do Brasil^(11)^, incluindo os serviços de diálise. Além disso, como arcabouço legislativo, destacam-se a Política Nacional de Atenção ao Portador de Doença Renal^(12)^, as linhas do cuidado para a pessoa com DRC^(13)^ e para pacientes adultos com DRC^(14)^, e o documento da Agência Nacional de Vigilância Sanitária, que institui nos serviços de diálise a cultura de SP, o gerenciamento de riscos e o estímulo às boas práticas focadas na qualidade e na segurança^(15)^. Essa base legal contempla ações fundamentais para prevenção, mitigação e tratamento das DRC, sendo a Atenção Primária em Saúde a gestora dos fluxos do paciente com fator de risco e com DRC^(12–15)^.

A equipe multidisciplinar dos serviços de diálise deve desenvolver ações em prol da SP, tais como: implementação de diretrizes, protocolos e estratégias de prevenção que direcionem o cuidado a fim de mitigar os riscos e promover a formação continuada dos profissionais^(16)^. No entanto, observam-se a falta de apoio da gestão e a baixa adesão dos profissionais da assistência^(17)^. Isso é reflexo da cultura organizacional, a qual orienta os profissionais^(18)^. Torna-se, portanto, fundamental compreender a cultura e o clima organizacional voltado para a SP nos serviços de diálise.

A cultura organizacional compreende valores e crenças intrínsecas, muitas vezes não percebidas, por se tratarem de fenômenos profundos dentro das organizações. Contudo, manifesta-se pelo clima organizacional em um determinado tempo, sofrendo influência de lideranças e de mudanças tanto internas como externas^(19)^. A cultura organizacional voltada para a SP é definida como o conjunto de valores e comportamentos dos profissionais que atuam no serviço, sendo preditiva da qualidade da assistência e da SP. Já o clima de SP consiste na maneira pela qual os profissionais percebem o ambiente de trabalho. Embora os conceitos apresentem suas singularidades, é comum o uso dos termos cultura e clima de SP como sinônimos na literatura, uma vez que possuem desfechos semelhantes e que se intersectam^(20)^. Dessa forma, optou-se por considerar ambos os termos neste estudo.

Para uma avaliação sistemática da percepção da cultura e do clima organizacional para SP são utilizados instrumentos específicos. No contexto da avaliação do clima de SP, destaca-se o *Safety Attitudes Questionnaire* (SAQ)^(21)^. Já para a mensuração da cultura de SP, são amplamente utilizados o *Medical Office Survey on Patient Safety* Culture (MOSPC)^(22)^ e o *Hospital Survey on Patient Safety Culture* (HSOPSC)^(23)^, os quais permitem uma análise minuciosa das práticas e percepções relacionadas à SP nas instituições de saúde. Esses instrumentos desempenham um papel crucial na coleta de dados confiáveis e no aprimoramento contínuo das estratégias de segurança em organizações de saúde. Eles capturam a percepção dos profissionais sobre o aprendizado organizacional decorrente de erros, a liberdade para notificar EA, o comprometimento da gestão com a SP e outros fatores que impactam direta ou indiretamente a cultura organizacional. Além disso, possibilitam a identificação de fragilidades, fortalezas e oportunidades de melhoria nos serviços de saúde.

A temática ainda é incipiente e pouco discutida na literatura^(24)^, incluindo a especificidade dos serviços dialíticos. Avaliar a cultura e o clima organizacional para SP nos serviços de saúde sob a percepção da equipe multidisciplinar contribui positivamente para a adoção de práticas seguras dentro dos serviços, uma vez que permite construir um plano de ação para melhorias contínuas do cuidado prestado, bem como efetuar o monitoramento dos efeitos das intervenções realizadas^(25,26)^. Nesse contexto, justifica-se o mapeamento e o agrupamento das evidências científicas publicadas acerca do assunto, que podem ainda fomentar o desenvolvimento de novos estudos e fortalecer a cultura e o clima organizacional para SP nos serviços de diálise. Diante do exposto, o objetivo do estudo foi mapear a literatura científica sobre a avaliação da cultura e do clima organizacional para SP em serviços de diálise renal na perspectiva da equipe multidisciplinar.

## MÉTODO

### Tipo de Estudo

Trata-se de uma revisão de escopo conduzida segundo a metodologia JBI^(27)^. O protocolo da revisão foi registrado na *Open Science Framework* (OSF): https://doi.org/10.17605/OSF.IO/EK93B ^(28)^. Para o direcionamento da escrita foram seguidas as recomendações do *Preferred Reporting Items for Systematic reviews and Meta-Analyses extension for Scoping Reviews* (PRISMA- ScR)^(29)^, específico para revisões de escopo.

### Questão Norteadora

A questão norteadora do estudo foi “Quais estudos disponíveis na literatura avaliaram a cultura e o clima organizacional para SP em serviços de diálise na perspectiva da equipe multidisciplinar?” A pergunta foi estruturada com base no acrônimo PCC- População, Conceito e Contexto: P- população (equipe multidisciplinar); C- conceito (a cultura e o clima organizacional para SP); C- contexto (serviços de diálise).

### Critérios de Seleção

Foram incluídos estudos que avaliaram a cultura e o clima organizacional para SP por meio de instrumentos de medida nos serviços de diálise sob a perspectiva da equipe multidisciplinar, disponíveis na íntegra, de qualquer área geográfica, nas bases de dados e na literatura cinzenta. Não houve recorte temporal nem restrição de idiomas. Foram excluídos estudos de revisão, pesquisas metodológicas, comentários, relatórios institucionais, resumos de eventos, manuais técnicos, cartas ao editor, relatos de casos e resenhas. Esses tipos de documentos não apresentaram dados empíricos primários ou resultados de avaliação necessários para identificar, descrever ou analisar a cultura e o clima organizacional. Assim, foram considerados inadequados para responder à questão de pesquisa proposta nesta revisão de escopo.

### Fontes de dados e Estratégias de Busca

As estratégias de busca foram delineadas com o auxílio de um profissional bibliotecário. A busca foi realizada em 20 de maio de 2024 nas bases de dados e nos repositórios institucionais Literatura Latino-Americana e do Caribe em Ciências da Saúde (LILACS), Base de Dados Enfermagem (BDENF), Índice Bibliográfico Espanhol de Ciências de Saúde (IBECS) e ColecionaSUS via Biblioteca Virtual em Saúde (BVS), *Medline* via *PubMed*, *Cochrane Library, Scopus, Web of Science* e *Embase*. As bases de dados foram acessadas por meio do Portal de Periódicos da CAPES, por meio da Comunidade Acadêmica Federada (CAFe). Foi realizada também a busca na literatura cinzenta no *Google Scholar*, no Catálogo de Teses e Dissertações da Coordenação de Aperfeiçoamento de Pessoal de Nível Superior (CAPES) e nos Repositórios das Universidades (UFC, UFAL, UNB, USP e UFMG). Esses repositórios institucionais foram selecionados devido à qualidade e relevância do conteúdo, bem como ao papel significativo das instituições na produção e disseminação de conhecimento, contribuindo para o avanço da ciência aberta e para a promoção de pesquisas inovadoras. As estratégias de busca foram adaptadas de acordo com as necessidades das bases de dados pesquisadas.

Para a seleção dos descritores foram utilizados DeCS (Descritores em Ciências da Saúde) /MeSH (*Medical Subject Headings*), com exceção da *Embase*, para a qual foi utilizado o Emtree. Todos os descritores incluídos na estratégia de busca foram termos controlados, ou seja, extraídos do DeCS/MeSH ou Emtree. Os operadores booleanos utilizados foram OR e AND. O operador OR aplicado para encontrar registros contendo um dos termos separados (ex.: *hemodialysis or dialysis*) e o AND para encontrar registros contendo todos os termos especificados (ex.: *hemodialysis and ‘patient safety*’), conforme apresentado no Quadro 1.

**Quadro 1:** Termos e estratégias de busca aplicadas a cada base de dados – Belo Horizonte, MG, Brasil, 2024.

Bases de dados e data da busca	Estratégias de busca	Número de estudos recuperados
LILACS via BVS 20 de maio de 2024	(“*Renal Dialysis*” OR “*Diálisis Renal*” OR “Diálise Renal” OR “Diálise Extracorpórea” OR “Hemodiálise” OR “*Dialysis*” OR “*Diálisis*” OR “Diálise” OR “*Hemodialysis Units Hospital*” OR “*Unidades de Hemodiálisis en Hospital*” OR “Unidades Hospitalares de Hemodiálise” OR “Unidades Hospitalares de Diálise Renal” AND (“*Patient Safety*” OR “*Seguridad del Paciente*” OR “Segurança do Paciente” OR “*Organizational Culture*” OR “Cultura Organizacional” OR “Cultura Organizacional” OR “Cultura Corporativa” OR “*Safety Management*” OR “*Administración de la Seguridad*” OR “Gestão da Segurança”) AND (db:(“LILACS” OR “IBECS” OR “BDENF” OR “colecionaSUS”) AND la:(“en” OR “es” OR “pt”))	96
MEDLINE via PUBMED 20 de maio de 2024	(“*Renal Dialysis*” OR “*Dialysis*” OR “*Hemodialysis Units Hospital*”) AND (“*Patient Safety*” OR “*Organizational Culture*” OR “*Safety Management*”)	597
*Cochrane Library* 20 de maio de 2024	(“*Renal Dialysis*” OR “*Dialysis*” OR “*Hemodialysis Units Hospital*”) AND (“*Patient Safety*” OR “*Organizational Culture*” OR “*Safety Management*”)	102
*Scopus* 20 de maio de 2024	(“*Renal Dialysis*” OR “*Dialysis*” OR “*Hemodialysis Units Hospital*”) AND (“*Patient Safety*” OR “*Organizational Culture*” OR “*Safety Management*”)	1745
*Web of Science* 20 de maio de 2024	(“*Renal Dialysis*” OR “*Dialysis*” OR “*Hemodialysis Units Hospital*”) AND (“*Patient Safety*” OR “*Organizational Culture*” OR “*Safety Management*”)	389
*Embase* 20 de maio de 2024	*hemodialysis* or *dialysis* AND *‘patient safety’*	2708
**Literatura cinzenta e data da busca**	**Estratégias de busca**	**Número de estudos recuperados**
*Google Scholar* 20 de maio de 2024	(Percepção) AND (“Cultura de segurança do paciente” OR “Clima de segurança do paciente”) AND (“Serviços de diálise”)	8000
Catálogo de teses e dissertações da CAPES 20 de maio de 2024	(Percepção) AND (“Cultura de segurança do paciente” OR “Clima de segurança do paciente”) AND (“Serviços de diálise”)	0
Repositórios (UFC, UFAL, UNB, USP, UFMG) 20 de maio de 2024	(Percepção) AND (“Cultura de segurança do paciente” OR “Clima de segurança do paciente”) AND (“Serviços de diálise”)	66

### Seleção dos Estudos

Dois pesquisadores selecionaram os estudos de forma independente e um terceiro avaliou as divergências até o consenso final. Utilizou-se o gerenciador de referências *Endnote*, onde foi realizada a exclusão das duplicatas. A partir disso, a lista de referências foi exportada para o gerenciador bibliográfico *Rayyan Qatar Computing Research Institute (Rayyan* QCRI); o processo de seleção dos estudos ocorreu em três etapas: análise dos títulos, leitura de resumos e leitura dos estudos na íntegra. Essas etapas foram norteadas pelos critérios de inclusão e exclusão. O mesmo processo ocorreu para a seleção dos artigos da literatura cinzenta, sendo que a busca manual nas listas de referências dos estudos incluídos foi analisada como estratégia para identificar produções científicas que não foram recuperadas anteriormente.

### Extração e Análise de Dados

Um banco de dados foi elaborado pelos pesquisadores a partir do programa *Microsoft Excel®* versão 2016, para extração dos seguintes dados: autor/ano de publicação; título; país de realização do estudo; objetivos; amostra; taxa de adesão dos participantes; população analisada; local do estudo; instrumento utilizado para avaliação da cultura ou do clima organizacional para SP; nível de cultura ou clima de SP; fortalezas; fragilidades e oportunidades de melhoria para cultura e clima organizacional para SP nos serviços de diálise.

Os dados extraídos e as principais características dos artigos selecionados foram compilados no Quadro 2. Para analisar e categorizar os resultados dos estudos que utilizaram o HSOPSC^(23)^ e o MOSPC^(22)^ e suas versões adaptadas foram utilizados os critérios de avaliação propostos pela *Agency for Healthcare Research and Quality* (AHRQ)^(23)^: áreas consideradas fortalezas ou fortes são aquelas que possuem 75% ou mais de porcentagens de respostas positivas (PRP) da equipe multidisciplinar, e as áreas consideradas frágeis ou com fragilidades quando as PRP forem iguais ou inferiores a 50%. Áreas avaliadas com <75% e >50% são consideradas como oportunidades de melhorias para cultura e clima organizacional para SP^(23)^. Já para a análise dos instrumentos SAQ versão adaptada^(30)^ e *Safety Climate Survey* (SCS)^(31)^, os autores consideraram áreas de fortalezas ou fortes com 75% ou mais de PRP e as áreas frágeis ou com fragilidades quando as PRP forem inferiores a 75%^(30,31)^.

**Quadro 2:** Caracterização dos estudos incluídos na revisão de escopo (n = 12) – Belo Horizonte, MG, Brasil, 2024.

Autor, ano e país	Amostra e taxa de adesão	Objetivos dos estudos	População	Local do serviço
Hoffmann et al.^(32)^ (2023); Brasil	n = 56 (%) = 71,8	Avaliar a cultura de SP na perspectiva da equipe de enfermagem em serviços de diálise durante a pandemia de COVID-19.	Enfermeiros, técnicos e auxiliares de enfermagem.	Três serviços de diálise de Minas Gerais
Rodrigues et al.^(24)^ (2023); Brasil	n = 134 (%) = 86	Avaliar os fatores associados à cultura de SP em diálise durante a pandemia de COVID-19.	Enfermeiros, técnicos e auxiliares de enfermagem, assistentes sociais, nutricionistas, psicólogos, farmacêuticos e o setor administrativo.	Três serviços de diálise de Minas Gerais
Silva^(33)^ (2019); Brasil	n = 32 (%) = 89	Avaliar a cultura de SP dos profissionais em um serviço de hemodiálise de hospital de ensino.	Enfermeiros, técnicos e auxiliares de enfermagem, médicos, setor administrativo, nutricionistas, áreas da limpeza e higienização, psicólogos, assistentes sociais, recepcionistas e técnicos de água.	Hospital Universitário da Bahia
Millson; Hackbarth; Benard^(34)^ (2019); EUA	n = 99 (%) ≥80	Avaliar as percepções da equipe de hemodiálise sobre a cultura de SP, implementar o programa de segurança da AHRQ e promover o aumento da adesão da equipe aos padrões de prevenção de infecção.	Enfermeiros e auxiliares e técnicos de enfermagem	Seis clínicas de diálise ambulatorial de Nova Iorque
Grilo^(35)^ (2018); Portugal	n = 108 (%) = 73	Analisar a percepção dos profissionais de saúde sobre a cultura de SP; comparar as percepções entre as diferentes categorias profissionais; e identificar áreas para melhoria da cultura de SP.	Enfermeiros, auxiliares e técnicos de enfermagem, médicos e administradores	Clínica de hemodiálise na região de Lisboa
Izquierdo et al.^(36)^ (2018); Espanha	n = 111 (%) = 85	Determinar a frequência das percepções e atitudes dos profissionais em centros de hemodiálise em relação à SP; e identificar pontos fortes e oportunidades de melhoria para a SP.	Enfermeiros, auxiliares de enfermagem e outras categorias	Seis centros de hemodiálise de Castela-Mancha
Dantas^(37)^ (2018); Brasil	n = 38 (%) = 100	Avaliar a cultura de SP em unidades de hemodiálise na perspectiva da equipe de enfermagem; e quantificar os eventos relatados por essa equipe.	Enfermeiros, técnicos e auxiliares de enfermagem	Duas unidades hospitalares de hemodiálise no Ceará
Davis et al.^(38)^ (2016); EUA	n = 581 (%) = 97	Avaliar as percepções da equipe multiprofissional sobre a cultura de SP em clínicas de hemodiálise.	Setor administrativo, médicos, nutricionista, assistente social, enfermeiros e técnicos de enfermagem	Clínicas de hemodiálise em duas cidades dos EUA
Ulrich; Kear^(39)^ (2015); EUA	n = 929 (%) = 95	Analisar as percepções dos enfermeiros nefrológicos sobre SP e sua cultura, considerando a unidade de trabalho, o ambiente organizacional e o papel desempenhado.	Enfermeiros	Associação Americana de Enfermeiras Nefrologistas
Ulrich; Kear^(40)^ (2014); EUA	n = 929 (%) = 95	Descrever os resultados da cultura de SP em ambientes de prática de enfermagem nefrológica; e comparar os dados da cultura de SP obtidos com os dados publicados pelo AHRQ.	Enfermeiros	Associação Americana de Enfermeiras Nefrologistas
Taher et al.^(41)^ (2014); Arábia Saudita	n = 509 (%) = 65	Avaliar o clima de SP percebido por enfermeiros e médicos em unidades de diálise na Arábia Saudita.	Enfermeiros e médicos	Cinco unidades de diálise em três cidades na Arábia Saudita
Di Benedetto et al.^(42)^ (2011); Itália	n = 346 (%) = 89	Avaliar o nível do clima de SP na rede de clínicas de diálise e a adesão da equipe de saúde à execução dos procedimentos- padrão para aprimoramento da segurança no serviço de diálise.	Enfermeiros, assistentes do cuidado e médicos	Trinta e três centros de diálise da *Fresenius Medical Care* na Itália

SP: Segurança do Paciente; COVID-19: *Coronavirus Disease* 2019; AHRQ: *Agency for Healthcare Research and Quality*; EUA: Estados Unidos da América.

Os estudos selecionados foram organizados por ano de publicação, e as áreas de fortalezas, oportunidades de melhoria e de fragilidades foram apresentadas juntamente com o instrumento de mensuração utilizado em formato de quadro.

Todos os pesquisadores participaram da interpretação e síntese dos dados dos artigos, utilizando a discussão narrativa para associar os resultados tabulados com o objetivo e a pergunta de pesquisa norteadora.

## RESULTADOS

Foram encontrados, nas bases de dados pesquisadas, 5.637 estudos no total. Após a remoção de 2.476 duplicatas, 3.161 estudos permaneceram. Destes, 3.123 foram excluídos após a leitura dos títulos e resumos. Não foi selecionada nenhuma evidência após a busca manual na lista das referências dos estudos incluídos. Dos 38 estudos selecionados para leitura na íntegra, excluíram-se 29 mediante justificativas, o que resultou na inclusão de nove estudos. Na literatura cinzenta foram identificados 8.066 estudos. Após a leitura dos títulos e resumos, 7.792 estudos foram excluídos por não responderem à questão norteadora e aos critérios de inclusão dessa revisão de escopo. Sendo assim, 274 foram avaliados para elegibilidade, dos quais 271 foram excluídos mediante justificativas, o que resultou na inclusão de três estudos na amostra. Por fim, 13.703 estudos foram encontrados, 312 foram lidos na íntegra e 12 foram selecionados para compor a amostra final. Todo esse processo foi descrito no fluxograma PRISMA ScR (Figura 1).

**Figura 1:** Fluxograma PRISMA-ScR de seleção dos estudos para inclusão na revisão de escopo – Belo Horizonte, MG, Brasil, 2024.

Os 12 estudos^(24,32–42)^ incluídos nessa revisão de escopo apresentaram abordagem metodológica quantitativa e delineamento transversal, cujos objetivos foram detalhados no Quadro 2. O período de publicação foi entre 2011 e 2023 e todos desenvolvidos em serviços de diálise públicos, privados ou filantrópicos. A maioria das pesquisas foi de origem norte-americana e brasileira (quatro estudos de cada, correspondendo a 33,4% do total em ambos os casos). A taxa de adesão dos participantes aos estudos variou entre 65% e 100%, com média de 85,5%. No âmbito da equipe multidisciplinar, a composição dos participantes divergiu entre os estudos analisados; dois estudos americanos^(39–40)^ avaliaram exclusivamente a percepção de enfermeiros nefrológicos (Quadro 2).

No que concerne aos instrumentos de avaliação, a maioria (n = 9; 75%) utilizou instrumentos para avaliar a cultura organizacional para SP, dentre os quais o HSOPSC^(23)^ foi o mais referenciado^(24,32–36,38–40)^. Os demais (n = 3; 25%) empregaram instrumentos para mensurar o clima organizacional para SP, com destaque para o SAQ^(37)^ e o SCS^(41–42)^.

As principais fortalezas identificadas foram relacionadas ao trabalho em equipe nos serviços de diálise^(35,36,38–40)^. Seguindo-se a esta, destacaram-se o aprendizado organizacional e a melhoria contínua (treinamentos), bem como o *feedback* e a comunicação sobre erro, presentes em 75% dos estudos (n = 9)^(24,33–36,38–41)^. Três estudos internacionais (25%)^(38–40)^ apontaram a importância de ações para o controle de infecção nas práticas nefrológicas como áreas fortalecidas para cultura e clima organizacional para SP nos serviços de diálise (Quadro 3).

**Quadro 3:** Dimensões da cultura e do clima organizacional para segurança do paciente (n = 12) – Belo Horizonte, MG, Brasil, 2024.

Artigos	Instrumento de avaliação da cultura e do clima organizacional para SP	Categorias	Dimensões dos instrumentos de avaliação da cultura e do clima organizacional para SP
Hoffman et al.^(32)^ (2023); Brasil	HSOPSC adaptado	Fortalezas	Expectativas e ações de promoção de segurança da direção/supervisão/gerentes da unidade/serviço (78%)
		Oportunidades de melhoria	Aprendizado organizacional/melhoria contínua (69%) *Feedback* e comunicação sobre erro (63%) Trabalho em equipe dentro das unidades/serviço (63%) Abertura da comunicação (55%)
		Fragilidades	Transferências internas e passagens de plantão (49%) Dimensionamento de pessoal (45%) Percepção geral de SP (45%) Apoio da gestão hospitalar para a SP (48%) Trabalho em equipe entre as unidades (45%) Frequência de eventos adversos notificados (43%) Respostas não punitivas aos erros (22%)
Rodrigues et al.^(24)^ (2023); Brasil	HSOPSC adaptado	Fortalezas	Aprendizado organizacional/melhoria contínua (86%) Expectativas e ações de promoção de segurança da direção/supervisão/gerentes da unidade/serviço (77%)
		Oportunidades de melhoria	Trabalho em equipe dentro das unidades/serviço (73%) Apoio da gestão hospitalar para a SP (64%) *Feedback* e comunicações sobre erro (60%) Frequência de eventos adversos notificados (59%) Transferências internas e passagens de plantão (57%) Abertura da comunicação (56%) Trabalho em equipe entre as unidades (56%) Percepção geral de SP (54%)
		Fragilidades	Dimensionamento de pessoal (49%) Respostas não punitivas aos erros (29%)
Silva^(33)^ (2019); Brasil	HSOPSC adaptado	Fortalezas	Aprendizado organizacional e melhoria contínua (84%)
		Oportunidades de melhoria	Trabalho em equipe dentro das unidades/serviço (74%) Dimensionamento de pessoal (58%)
		Fragilidades	Respostas não punitivas aos erros (47%) *Feedback* e comunicação sobre erro (46%) Apoio da gestão hospitalar para a SP (45%) Frequência de eventos adversos notificados (44%) Abertura da comunicação (38%) Expectativas e ações de promoção de segurança da direção/supervisão/gerentes da unidade/serviço (35%) Trabalho em equipe entre as unidades (32%) Percepção geral de SP (26%) Transferências internas e passagens de plantão (19%)
Millson; Hackbart; Benard (2019)^(34)^; EUA	HSOPSC adaptado	Fortalezas	Aprendizado organizacional e melhoria Contínua (80%) Abertura da comunicação (77%)
		Oportunidades de melhoria	Apoio da gestão hospitalar para a SP (63%) Trabalho em equipe entre as unidades (57%)
		Fragilidades	Não mencionadas
Grilo^(35)^ (2018); Portugal	HSOPSC adaptado	Fortalezas	Trabalho em equipe dentro das unidades (81%) Aprendizado organizacional e melhoria contínua (77%) Apoio da gestão hospitalar para a SP (75%) Trabalho em equipe entre as unidades (75%)
		Oportunidades de melhoria	Percepção geral de SP (72%) Expectativas e ações de promoção de segurança da direção/supervisão/gerentes da unidade/serviço (67%) Transferências internas e passagens de plantão (61%) Dimensionamento de pessoal (55%) Frequência de eventos adversos notificados (58%)
		Fragilidades	*Feedback* e comunicação sobre erro (50%) Abertura da comunicação (50%) Respostas não punitivas aos erros (37%)
Izquierdo et al.^(36)^ (2018); Espanha	HSOPSC adaptado	Fortalezas	Trabalho em equipe dentro das unidades (86%) *Feedback* e comunicação sobre erro (76%) Expectativas e ações de promoção de segurança da direção/supervisão/gerentes da unidade/serviço (75%)
		Oportunidades de melhoria	Frequência de eventos adversos notificados (73%) Aprendizado organizacional e melhoria contínua (71%) Percepção geral da SP (70%) Abertura da comunicação (65%) Dimensionamento de pessoal (63%) Respostas não punitivas aos erros (63%) Transferências internas e passagens de plantão (62%) Apoio da gestão hospitalar para a SP (59%) Trabalho em equipe entre as unidades (55%)
		Fragilidades	Não mencionadas
Dantas^(37)^ (2018); Brasil	SAQ adaptado	Fortalezas	Satisfação no trabalho (86%) Clima de trabalho em equipe (82%) Condições de trabalho (79%)
		Oportunidades de melhoria	Não mencionadas
		Fragilidades	Clima de SP (72%) Percepção do estresse (67%)
Davis et al.^(38)^ (2016); EUA	HSOPSC adaptado	Fortalezas	Apoio da gestão hospitalar para a SP (88%) Quando há muita coisa a ser feita rapidamente, conseguimos trabalhar como uma equipe (86%) Gestores consideram a sugestão dos trabalhadores sobre as medidas para prevenir infecções associadas ao acesso vascular (84%) São discutidas maneiras de evitar a repetição dos erros na unidade (83%) Estamos ativamente modificando os protocolos e políticas para reduzir as infecções relacionadas ao acesso vascular em nossa instituição (82%) Depois de fazermos alterações para melhorar a SP, avaliamos sua eficácia (81%) Meu supervisor ou gerente oferece *feedback* positivo quando identifica que o trabalho foi realizado conforme os procedimentos estabelecidos para a SP (79%) Os trabalhadores se sentem à vontade para falar caso percebam algo que possa aumentar o risco de infecções relacionadas ao acesso vascular (76%) Erros podem levar a mudanças positivas (76%) Tratar-se mutualmente com respeito (75%)
		Oportunidades de melhoria	Não mencionadas
		Fragilidades	Não mencionadas
Ulrich; Kear^(39)^ (2015); EUA	HSOPSC e MOSPC adaptados	Fortalezas	Trabalho em equipe (87%) Treinamento da equipe (80%) Controle de infecção na diálise (76%) Liderança contínua - aprendizado organizacional (76%)
		Oportunidades de melhoria	Apoio da gestão hospitalar para a SP (74%) Expectativas e ações de promoção de segurança da direção/supervisão/gerentes da unidade/serviço (70%) Padronização de processos (69%) Percepção geral da SP (65%) Abertura da comunicação (64%) *Feedback* e comunicação sobre erro (63%) Suporte organizacional da liderança para SP (60%) Frequência dos eventos adversos notificados (55%) Respostas não punitivas aos erros (55%) Dimensionamento de pessoal (54%)
		Fragilidades	Não mencionadas
Ulrich; Kear^(40)^ (2014); EUA	HSOPSC adaptado	Fortalezas	Trabalho em equipe entre as unidades (81%) Controle de infecção (79%) Treinamento da equipe (78%)
		Oportunidades de melhoria	Aprendizado organizacional e melhoria contínua (73%) Expectativas e ações de promoção de segurança da direção/supervisão/gerentes da unidade/serviço (72%) Apoio da gestão hospitalar para a SP (67%) Percepção geral da SP (65%) Padronização de processos (62%) Abertura da comunicação (60%) *Feedback* e comunicação sobre erro (59%) Dimensionamento de pessoal (57%) Suporte organizacional da liderança para a SP (56%) Respostas não punitivas aos erros (55%) Frequência dos eventos adversos notificados (55%)
		Fragilidades	Transferências internas e passagens de plantão (33%)
Taher et al.^(41)^ (2014); Arábia Saudita	SCS	Fortalezas	A passagem de informações no início do turno é importante para a SP (85%) O pessoal assume a responsabilidade pela SP (84%) A SP é considerada uma prioridade (83%) A comunicação de informações é frequente neste ambiente (81%) Eu conheço com propriedade os canais para tirar dúvidas sobre questões de SP (79%) Eventos adversos estão frequentemente associados com múltiplas falhas no sistema (78%) Os médicos e os enfermeiros líderes da minha área clínica me escutam e consideram minhas preocupações (77%) Eu me sinto encorajado pelos meus colegas para comunicar qualquer preocupação que eu possa ter com a SP (76%) Mais esforços para SP foram realizados em comparação com o ano passado (76%)
		Oportunidades de melhoria	Não mencionadas
		Fragilidades	A liderança está nos conduzindo a atuar como uma instituição centrada na SP (74%) Erros médicos são adequadamente tratados (69%) A cultura de SP desta área clínica propicia o aprendizado baseado nos erros (68%) Eu me sentiria seguro em ser tratado nesta unidade (67%) Eu recebo um *feedback* apropriado sobre meu desempenho (64%) A gestão não ignora questões de SP em prol da produtividade (62%) Minhas sugestões sobre SP são consideradas pela gestão (68%)
Di Benedetto et al.^(42)^ (2011) – Itália	SCS	Fortalezas	A liderança está nos conduzindo a atuar como uma instituição centrada na SP (93%) Os médicos e os enfermeiros líderes da minha área clínica me escutam e consideram minhas preocupações (83%) Sou encorajado pelos meus colegas a relatar quaisquer preocupações que possa ter sobre a SP (85%) A cultura de SP desta área clínica propicia o aprendizado baseado nos erros (82%)
		Oportunidades de melhoria	Não mencionadas
		Fragilidades	Não mencionadas

SP: Segurança do Paciente; HSOPSC: *Hospital Survey on Patient Safety Culture*; SAQ: *Safety Attitudes Questionnaire*; MOSPC: *Medical Office Survey on Patient Safety Culture*; SCS: *Safety Climate Scale*.

O tipo de gestão dos serviços também foi avaliado como fator que influencia a percepção dos profissionais. Dois estudos (17%) brasileiros^(24,32)^ identificaram os fatores laborais predisponentes à cultura e ao clima organizacional para SP nas práticas assistenciais nefrológicas. Um dos estudos^(24)^ identificou mais áreas fortes nos serviços de diálise com gestão privada e filantrópica; as áreas frágeis, por sua vez, foram mais frequentemente relatadas no serviço de gestão pública, conforme a percepção da equipe multidisciplinar. No entanto, outro estudo^(32)^ apontou que a equipe de enfermagem do serviço de diálise público apresentou melhor percepção da cultura e do clima organizacional para SP quando comparada às equipes dos serviços de diálise filantrópicos e privados.

Na maioria dos estudos (n = 8; 67%), a percepção geral da cultura e do clima organizacional para SP foi avaliada pelos participantes como positiva^(24,32,35,38–42)^. Todavia, dois estudos brasileiros (n = 2; 17%) avaliaram a percepção geral como predominantemente negativa^(33,37)^.

## DISCUSSÃO

A partir dos resultados identificou-se que os estudos mais recentes sobre a avaliação da cultura e do clima organizacional para SP nos serviços de diálise foram publicados entre os anos de 2019 e 2023, dos quais três eram brasileiros^(24,32,33)^ e um americano^(34)^. Destaca-se que dois estudos foram desenvolvidos durante a pandemia de *Coronavirus Disease* 2019 (COVID-19) no Brasil, um com foco na percepção da equipe de enfermagem^(32)^ e o outro na da equipe multidisciplinar^(24)^. Assim, diante das inúmeras dificuldades vivenciadas no contexto pandêmico para a produção científica nesta área, é imprescindível que gestores e formuladores de políticas públicas promovam a construção e a implementação de estratégias para melhorar a cultura e o clima organizacional para SP nos ambientes de práticas nefrológicas^(16)^.

A maioria dos estudos foi conduzida em países desenvolvidos, nos quais a cultura e o clima organizacional para SP foram mais bem avaliados em comparação aos estudos realizados em países de baixa ou média renda^(38,42)^. O Brasil foi o único país da América Latina onde foi possível encontrar estudos sobre a temática. Diante desse cenário, suscita-se a discussão sobre a interferência negativa das desigualdades socioeconômicas e geográficas na percepção da cultura e do clima organizacional para SP por parte da equipe multidisciplinar dos serviços de diálise. Similarmente, estudos evidenciaram que as diversas desigualdades interferiram negativamente no acesso aos serviços de saúde e na qualidade da assistência prestada, configurando-se em um problema de saúde pública^(43,44)^.

Avaliar a cultura e o clima organizacional para SP deve ser prioridade de gestores e profissionais nos serviços de saúde, pois isso possibilita a identificação e a mensuração de condições organizacionais que podem propiciar o aumento de EA e impactar a qualidade do cuidado ofertado, além de estimular o desenvolvimento de estratégias de melhoria da SP na Rede de Atenção em Saúde^(45)^. Os instrumentos de avaliação da cultura e do clima de SP podem direcionar a compreensão e a transformação das atitudes e do comportamento relacionados à SP, a partir da percepção dos profissionais de uma organização de saúde^(45)^.

As áreas de maior força para a cultura e o clima organizacional para SP, que se destacaram, foram: aprendizado organizacional e melhoria contínua; *feedback* e comunicação sobre erro; e trabalho em equipe. A cultura e o clima organizacional positivos para SP podem direcionar o comportamento da equipe para a prestação de um cuidado seguro. Por isso, as áreas de força destacadas nos estudos devem ser prioridades nos serviços de saúde. Assim, contribui-se para o encorajamento dos profissionais na realização da análise de erros e EA, com o objetivo de aprimorar continuamente a SP nos serviços de saúde^(46)^.

Especificamente em serviços de diálise, os profissionais reconhecem que o fortalecimento da cultura e do clima organizacional para SP propicia o aprimoramento e o desenvolvimento de competências essenciais para uma prática assistencial segura^(24)^. Nessa perspectiva, os serviços de saúde devem substituir a cultura punitiva do erro por uma cultura de aprendizagem, que fomenta a comunicação aberta dos incidentes sem repressão ou punição^(47)^.

A força do trabalho em equipe conecta o conhecimento e as habilidades de diferentes pessoas com uma única finalidade: a assistência ao paciente^(45)^. Todavia, o trabalho da equipe multidisciplinar em saúde apresenta desafios como a existência de conflitos, o individualismo e a hierarquização das relações^(48)^. É importante que os profissionais adotem medidas para um desenvolvimento adequado do trabalho em equipe, com foco na contribuição para a qualidade e o acesso aos serviços de saúde^(49)^.

Estudos internacionais^(38–40)^ evidenciaram a prevenção de infecções em serviços de diálise para promover a melhoria da cultura e do clima organizacional para SP; mediante isso, o risco de infecção é considerado uma das principais causas de mortalidade e internação hospitalar em pacientes em tratamento dialítico^(5)^. Estratégias como a implementação da análise investigativa (causa-raiz) para determinar problemas de sistemas e processos são necessárias para diminuir e mitigar os riscos e os EA nos serviços de diálise^(16)^. Além disso, ações específicas na área da higienização das mãos e cuidados com cateteres venosos centrais devem ser priorizadas para controle efetivo de infecção^(16,26)^.

Estudos brasileiros^(24,32,33,37)^ apresentaram áreas relativas à percepção geral da cultura e do clima organizacional para SP como uma fragilidade ou oportunidade de melhoria. Isso pode estar associado aos desafios profissionais dos serviços públicos brasileiros, como a alta demanda de atendimento e a falta de maiores investimentos em recursos humanos e tecnológicos^(50)^.

Três estudos (25%) evidenciaram o dimensionamento de pessoal e a transição/transferência/passagem de plantão entre unidades e serviços de diálise como fragilidades^(24,32,40)^. Entretanto, quatro estudos (33%) identificaram essas áreas como oportunidades de melhoria para cultura e clima organizacional para SP^(33,35,36,39)^. Em dois estudos (17%), as condições de trabalho foram consideradas áreas fortes^(37,41)^. Ressalta-se que o dimensionamento da equipe nos serviços de diálise no Brasil é previsto pela Resolução da Diretoria Colegiada (RDC) nº 11/2014, que também estabelece requisitos de boas práticas de funcionamento para os serviços de diálise^(51)^.

Estudo brasileiro com a equipe de enfermagem evidenciou que as condições inadequadas de trabalho, decorrentes do ambiente e das funções exercidas, impactam negativamente na percepção da cultura e do clima organizacional para SP^(52)^. O ambiente de trabalho gera desgaste físico e mental devido à ausência de reconhecimento profissional, à falta de motivação, à falta de insumos, à remuneração inadequada, à carga horária de trabalho excessiva, ao dimensionamento incorreto de profissionais, à jornada noturna e à insatisfação dos profissionais^(52)^.

Destaca-se que a passagem de plantão é o momento em que as informações mais importantes relacionadas à evolução dos pacientes são discutidas pela equipe multidisciplinar, o que pode contribuir para a continuidade da assistência e a garantia da SP, desde que seja desenvolvida por meio de uma comunicação efetiva^(53)^. Para tal finalidade, é imprescindível a padronização mediante protocolos e ferramentas de comunicação que garantam a continuidade das ações, além do incentivo às mudanças de atitudes e do fortalecimento da cultura e do clima organizacional^(52)^.

O apoio dos gestores e da liderança às preocupações, demandas e sugestões da equipe multidisciplinar atuante nos serviços de diálise foi destacado como dimensão frágil ou como oportunidade de melhoria em 75% (n = 9) dos estudos^(24,32–34,36,39–42)^. A participação da liderança no acolhimento das demandas e sugestões da equipe multidisciplinar é de extrema importância na promoção da SP. Estudo iraniano conduzido em hospitais aponta que gestores, líderes e supervisores devem promover ações em prol do desenvolvimento de valores, crenças e comportamento pessoal alinhado aos valores da cultura e do clima organizacional. O comprometimento do líder na identificação de problemas no processo de trabalho e no desenvolvimento de estratégias que subsidiem recursos e oportunidades de aprendizagem pode contribuir para a melhoria da prestação dos cuidados de saúde^(46)^.

Além disso, o modelo de gestão adotado pela organização de saúde pode influenciar na percepção dos profissionais em relação à cultura e ao clima organizacional para SP. Nesse sentido, é preciso que todos estejam comprometidos com a manutenção e promoção da cultura e do clima de SP. Sendo assim, a gestão possui papel essencial na melhoria da cultura e do clima organizacional para SP, na medida em que promove a organização de fluxos que melhoram a efetividade do trabalho em saúde^(54)^.

As dimensões da cultura e do clima organizacional para SP “frequência de EA notificados” e “respostas não punitivas aos erros” foram citadas como oportunidade de melhoria ou fragilidade em sete estudos (58%)^(24,32,33,35,36,39,40)^. A dimensão “EA são frequentemente associados com múltiplas falhas no sistema” foi considerada fortaleza em três (25%) pesquisas^(36,38,41)^. Evidencia-se, assim, que a existência de uma cultura punitiva e a sobrecarga de trabalho inibem os profissionais de realizar as notificações de EA. Esses resultados estão em consonância com outro estudo, no qual a cultura punitiva e a carga de trabalho excessiva também são consideradas empecilhos para a notificação de EA^(53)^. Nessa perspectiva, a subnotificação de EA deve ser trabalhada como oportunidade de melhoria, já que impede o desenvolvimento de ações para evitar a recorrência dos erros em saúde e o diagnóstico de riscos do serviço.

A influência da categoria profissional na percepção da cultura e do clima organizacional para SP foi analisada pelos estudos com resultados diferenciados. Em cinco artigos (42%), administradores/gestores/líderes apresentaram uma percepção geral mais positiva da cultura e do clima organizacional para SP quando comparados com o restante da equipe multidisciplinar de diálise^(24,35,38,40,42)^. Dois estudos (17%) identificaram que médicos, equipe multidisciplinar e setor administrativo relataram mais áreas de fortalezas quando comparados com a equipe de enfermagem^(24,38)^. Dois estudos brasileiros destacaram que os enfermeiros, quando comparados com médicos ou auxiliares e técnicos de enfermagem, apresentaram uma melhor avaliação da percepção da cultura e do clima organizacional para SP^(32,37)^. Estudo conduzido na Arábia Saudita também corrobora esse achado^(41)^. Em contrapartida, estudo brasileiro^(33)^ aponta que a percepção entre os auxiliares e técnicos de enfermagem foi mais bem avaliada em comparação com a dos enfermeiros e médicos dos serviços de diálise. Dois estudos americanos avaliaram a cultura e o clima organizacional para SP sob a perspectiva de 929 enfermeiros atuantes em serviços de diálise^(39,40)^. A percepção dos enfermeiros é considerada essencial, dada sua proximidade com os pacientes, função de liderança e papel central no controle de infecções e comunicação efetiva na equipe^(24,39,40)^.

Portanto, notou-se nos estudos influência da categoria profissional na percepção da cultura e clima organizacional para SP nos serviços de diálise. Contudo, não foi possível identificar um padrão de como essas influências ocorrem a partir dos estudos mapeados. Estudo internacional identificou que gestores e setor administrativo apresentaram uma percepção mais positiva da cultura e clima de SP quando comparados com os demais integrantes da equipe multidisciplinar^(55)^.

Nesse sentido, observou-se uma variabilidade na percepção da equipe multidisciplinar no cenário brasileiro. Um estudo identificou uma percepção mais positiva sobre a cultura e o clima organizacional para SP nos serviços de diálise pelos gestores e setor administrativo^(24)^. Dois estudos destacaram a percepção dos enfermeiros comparada à dos médicos e demais membros da equipe de enfermagem^(32,37)^. Por fim, um identificou uma melhor percepção entre os técnicos e auxiliares de enfermagem comparados com enfermeiros e médicos^(33)^. Contudo, desigualdades na percepção dos profissionais sobre cultura e clima organizacional para SP necessitam ser corrigidas por meio da sua valorização profissional e discussões acerca de práticas que incentivem o cuidado seguro^(56)^.

Além disso, é necessário que a temática seja abordada nas diretrizes curriculares nacionais e no plano político pedagógico da formação profissional na área da saúde durante o processo de ensino-aprendizagem, pois é essencial para o fortalecimento da cultura e do clima de SP, visto que os atuais estudantes serão os futuros profissionais dos serviços de saúde^(57)^, inclusive dos serviços de diálise.

A categoria profissional também influenciou a percepção da cultura e do clima organizacional para SP conforme o modelo de gestão dos serviços de diálise^(24,32)^. Tais diferenças podem ser justificadas por algumas hipóteses, já que instituições privadas recebem maior investimento de gestores em ações de melhorias nos processos de trabalho e na qualidade da assistência prestada devido à competitividade do mercado^(24)^. Em contrapartida, a equipe de enfermagem desenvolve habilidades e competências direcionadas à identificação de riscos que envolvem a SP devido à proximidade com o paciente durante a assistência prestada, quando comparados a outras categorias profissionais^(52)^. Por fim, o serviço de diálise público durante a pandemia teve treinamentos de fluxos assistenciais por se tratar de hospital-referência para tratamento de COVID-19, o que pode ter influenciado na percepção da equipe de enfermagem^(32)^.

Diante disso, os desfechos dos estudos mapeados evidenciaram que a cultura e o clima organizacional para SP nos serviços diálise são percebidos pela equipe multiprofissional como relativamente positivos ou neutros (oportunidades de melhoria). Entretanto, espera-se encontrar uma cultura e um clima organizacional para SP positiva em todos os serviços, uma vez que a consolidação deles é fundamental para aprimorar a qualidade da assistência. Para isso, é necessário que os gestores e líderes reconheçam a importância da comunicação para identificar as preocupações dos profissionais da assistência e dos pacientes^(52)^. Ademais, a colaboração da equipe multidisciplinar é indispensável para manter as fortalezas, aperfeiçoar as oportunidades de melhoria e reparar as fragilidades nas áreas da cultura e do clima organizacional para SP nos serviços de diálise.

Notou-se que a literatura acerca dessa temática é incipiente e precisa ser valorizada no contexto mundial, pois foram identificados poucos estudos que avaliassem a cultura e o clima organizacional de SP nos serviços de diálise sob a percepção da equipe multidisciplinar. Destaca-se como limitação o fato de que dois estudos incluídos^(39,40)^ abordaram exclusivamente a percepção de enfermeiros, o que pode restringir a generalização dos achados para toda a equipe multidisciplinar.

Outro ponto importante é a falta de consenso na literatura sobre o uso dos termos “cultura” e “clima organizacional” para SP, aspecto que dificultou o processo de análise de dados dos estudos desta revisão. Os pesquisadores minimizaram a dificuldade ao considerar ambos os termos, uma vez que estão intrinsecamente correlacionados e refletem na prática assistencial e gerencial dos serviços de diálise. No entanto, esta revisão contribuirá para a prática clínica e gerencial dos serviços de diálise na medida em que promove o aumento da compreensão sobre a cultura e o clima organizacional para SP sob a perspectiva da equipe multidisciplinar.

## CONCLUSÃO

As maiores fortalezas para cultura e clima organizacional para SP foram nas áreas de trabalho em equipe, aprendizado organizacional e melhoria contínua, *feedback* e comunicação sobre erro. As fragilidades e as oportunidades de melhoria envolveram o dimensionamento pessoal, a transição de plantão e transferências internas, o apoio da liderança, a notificação de eventos adversos e as respostas não punitivas aos erros.

A categoria profissional, a localização geográfica e o tipo de gestão dos serviços de diálise influenciaram a percepção da cultura e do clima organizacional para SP, e estas se apresentaram positivas na maioria dos estudos. Os achados desta revisão poderão auxiliar gestores, líderes e profissionais no planejamento estratégico do cuidado prestado nos serviços de diálise, a partir da avaliação da cultura e do clima organizacional para SP, com a finalidade de propor ações de melhoria contínua da SP e do cuidado em saúde. Assim, contribuirá no direcionamento de estudos futuros que promovam o fortalecimento da cultura e do clima de SP e das práticas nefrológicas seguras no âmbito da saúde pública brasileira. Os achados apontaram áreas de fragilidades, mas não apresentaram compreensão detalhada dos fatores que contribuem para tais resultados, o que suscita a necessidade de condução de novos estudos, a fim de obter maior caracterização de como a equipe multidisciplinar avalia e percebe a cultura e o clima organizacional para SP nos serviços de diálise renal, bem como uma possível generalização dos resultados.

## DISPONIBILIDADE DE DADOS

Todo o conjunto de dados que dá suporte aos resultados deste estudo foi disponibilizado e publicado no próprio artigo.

# Translation: Cultura y clima organizacional para la seguridad del paciente en servicios de diálisis: revisión del alcance

**DOI:** 10.1590/1980-220X-REEUSP-2024-0319es

**Authors:** Marília Alves Hoffmann, Aline Carrilho Menezes, Tatiana Aparecida Rodrigues, Larissa Assis Caputo Figueiredo, Aldenora Laísa Paiva de Carvalho Cordeiro, Helen Cristiny Teodoro Couto Ribeiro, Luciana Regina Ferreira da Mata

**Affiliations:** 1 Universidade Federal de Minas Gerais, Escola de Enfermagem, Programa de Pós-Graduação em Enfermagem, Belo Horizonte, MG, Brasil.; 2 Universidade Federal de São João Del-Rei, Divinópolis, MG, Brasil.; 3 Universidade Federal de São João Del-Rei, Programa de Pós-Graduação em Enfermagem, Divinópolis, MG, Brasil.; 4 Universidade Federal de Minas Gerais, Escola de Enfermagem, Departamento de Enfermagem Básica, Belo Horizonte, MG, Brasil.

**Keywords:** Cultura Organizacional, Seguridad del Paciente, Diálisis Renal, Grupo de Atención al Paciente

## Abstract

**Objetivo:**

Mapear la literatura científica sobre la evaluación de la cultura y el clima organizacional para la seguridad del paciente en servicios de diálisis renal desde la perspectiva del equipo multidisciplinario.

**Método:**

Revisión del alcance según la metodología JBI. Los artículos fueron extraídos de LILACS vía BVS, *Medline* a través de PubMed, *Biblioteca Cochrane*, *Scopus*, *Web de la ciencia*, *Base* y literatura gris (*Google Académico*, CAPES y repositorios universitarios). Todos los descriptores fueron términos controlados extraídos de DeCS/MeSH o Emtree. La pregunta guía fue: ¿qué estudios disponibles en la literatura evaluaron la cultura y el clima organizacional para la seguridad del paciente en servicios de diálisis desde la perspectiva del equipo multidisciplinario?

**Resultados:**

Se identificaron 13.703 estudios, 312 fueron leídos en su totalidad y 12 fueron incluidos. Las mayores fortalezas se encontraron en los dominios: trabajo en equipo, aprendizaje organizacional y mejora continua, *feedback* y comunicación de errores. Las debilidades y oportunidades de mejora incluyeron la dotación de personal, la transición de turnos, el apoyo del liderazgo, los informes de eventos adversos y las respuestas no punitivas a los errores. La categoría profesional y el tipo de gestión influyeron en la percepción del equipo de atención al paciente.

**Conclusión:**

Los hallazgos destacaron áreas de debilidad en la cultura y el clima organizacional para la seguridad del paciente en las prácticas de nefrología, pero no detallaron los factores causales, lo que requiere más estudios.

## INTRODUCCIÓN

La hemodiálisis es la principal terapia renal sustitutiva utilizada en el mundo, con una prevalencia del 89%^(1)^. En Brasil, los costos aumentan con la progresión de la enfermedad renal crónica (ERC) y el enfoque del Sistema Único de Salud se centra más en las terapias dialíticas, en detrimento de las medidas preventivas^(2)^. En 2022, Brasil registró aproximadamente 153 831 pacientes en terapia dialítica, de los cuales el 95,3% en hemodiálisis y el 4,7% en diálisis peritoneal, atendidos por 872 centros activos de diálisis crónica, de los cuales 243 participaron en el Censo Brasileño de Diálisis, con un 75,7% de carácter privado, el 15,6% filantrópicos y el 8,6% públicos^(3)^.

La terapia renal sustitutiva, ya sea por hemodiálisis o diálisis peritoneal, desempeña un papel esencial en el manejo de la ERC. Sin embargo, el aumento de la necesidad de tratamiento dialítico y las características de la ERC hacen que los pacientes sean más vulnerables a eventos adversos (EA) que pueden comprometer su salud y calidad de vida^(4)^. Los procesos infecciosos, el desplazamiento de la fístula arteriovenosa, la hipotensión intra-dialítica, las arritmias y la embolia gaseosa son los EA más frecuentes en la hemodiálisis^(5)^, lo que contribuye al aumento de la tasa de mortalidad^(6)^. Por otro lado, la diálisis peritoneal puede dar lugar a complicaciones infecciosas y mecánicas, como peritonitis y migración u obstrucción del catéter, que pueden afectar negativamente a la seguridad y la eficacia del tratamiento^(7)^. En 2019, 3,16 millones de personas murieron a causa de la ERC en todo el mundo^(8)^. En Brasil, entre 2009 y 2020, se observó una tendencia al alza en la tasa de mortalidad por ERC en ambos sexos, especialmente en el grupo de edad superior a 75 años, principalmente en las regiones del norte y noreste del país^(9)^.

Desde esta perspectiva, la asistencia sanitaria prestada en los servicios de diálisis debe basarse en los principios de la seguridad del paciente (SP). La SP se estructura en torno a actividades destinadas a promover estrategias que orienten la cultura organizativa de los servicios de salud y la reducción de los riesgos y daños evitables^(10)^. Instituido en 2013, el Programa Nacional de SP tiene como objetivo garantizar la calidad de la atención en todos los servicios de salud de Brasil^(11)^, incluidos los servicios de diálisis. Además, como marco legislativo, destacan la Política Nacional de Atención al Portador de Enfermedad Renal^(12)^, las líneas de atención a las personas con ERC^(13)^ y a los pacientes adultos con ERC^(14)^, y el documento de la Agencia Nacional de Vigilancia Sanitaria, que establece en los servicios de diálisis la cultura de la SP, la gestión de riesgos y el fomento de buenas prácticas centradas en la calidad y la seguridad^(15)^. Esta base legal contempla acciones fundamentales para la prevención, mitigación y tratamiento de la ERC, siendo la Atención Primaria en Salud la encargada de gestionar los flujos de pacientes con factores de riesgo y con ERC^(12–1 5)^.

El equipo multidisciplinario de los servicios de diálisis debe desarrollar acciones en favor de la SP, tales como: implementación de directrices, protocolos y estrategias de prevención que orienten la atención con el fin de mitigar los riesgos y promover la formación continua de los profesionales^(16)^. Sin embargo, se observa la falta de apoyo de la dirección y la baja adhesión de los profesionales de la asistencia^(17)^. Esto es un reflejo de la cultura organizacional, que orienta a los profesionales^(18)^. Por lo tanto, es fundamental comprender la cultura y el clima organizacional orientado a la SP en los servicios de diálisis.

La cultura organizacional comprende creencias y valores intrínsecos, a menudo no percibidos, ya que se trata de fenómenos profundos dentro de las organizaciones. Sin embargo, se manifiesta a través del clima organizacional en un momento determinado, bajo la influencia de los líderes y de los cambios tanto internos como externos^(19)^. La cultura organizacional orientada a la SP se define como el conjunto de valores y comportamientos de los profesionales que trabajan en el servicio, siendo predictiva de la calidad de la asistencia y de la SP. Por su parte, el clima de SP consiste en la forma en que los profesionales perciben el entorno de trabajo. Aunque los conceptos presentan sus singularidades, es común el uso de los términos cultura y clima de SP como sinónimos en la literatura, ya que tienen resultados similares y se entrecruzan^(20)^. Por lo tanto, se optó por considerar ambos términos en este estudio.

Para una evaluación sistemática de la percepción de la cultura y el clima organizacional para la SP se utilizan instrumentos específicos. En el contexto de la evaluación del clima de la SP, destaca el *Safety Attitudes Questionnaire* (SAQ)^(21)^. Para medir la cultura de la SP, se utilizan ampliamente la *Medical Office Survey on Patient Safety* Culture (MOSPC)^(22)^ y la *Hospital Survey on Patient Safety Culture* (HSOSPC)^(23)^, que permiten un análisis minucioso de las prácticas y percepciones relacionadas con la SP en las instituciones de salud. Estos instrumentos desempeñan un papel crucial en la recolección de datos confiables y en la mejora continua de las estrategias de seguridad en las organizaciones de salud. Capturan la percepción de los profesionales sobre el aprendizaje organizacional derivado de los errores, la libertad para notificar EA, el compromiso de la dirección con la SP y otros factores que impactan directa o indirectamente en la cultura organizacional. Además, permiten identificar debilidades, fortalezas y oportunidades de mejora en los servicios de salud.

El tema aún es incipiente y poco discutido en la literatura^(24)^, incluida la especificidad de los servicios de diálisis. Evaluar la cultura y el clima organizacional para la SP en los servicios de salud desde la percepción del equipo multidisciplinario contribuye positivamente a la adopción de prácticas seguras dentro de los servicios, ya que permite construir un plan de acción para la mejora continua de la atención prestada, así como realizar el seguimiento de los efectos de las intervenciones realizadas^(25,26)^. En este contexto, se justifica el mapeo y la agrupación de las evidencias científicas publicadas sobre el tema, que pueden fomentar el desarrollo de nuevos estudios y fortalecer la cultura y el clima organizacional para la SP en los servicios de diálisis. Ante lo expuesto, el objetivo del estudio fue mapear la literatura científica sobre la evaluación de la cultura y el clima organizacional para la SP en los servicios de diálisis renal desde la perspectiva del equipo multidisciplinario.

## MÉTODO

### Tipo de Estudio

Se trata de una revisión de alcance realizada según la metodología JBI^(27)^. El protocolo de la revisión se registró en el *Open Science Framework* (OSF): https://doi.org/10.17605/OSF.IO/EK93B ^(28)^. Para orientar la redacción, se siguieron las recomendaciones de los *Preferred Reporting Items for Systematic reviews and Meta-Analyses extension for Scoping Reviews* (PRISMA- ScR)^(29)^, específicos para revisiones de alcance.

### Pregunta Orientadora

La pregunta orientadora del estudio fue «¿Qué estudios disponibles en la literatura evaluaron la cultura y el clima organizacional para la SP en los servicios de diálisis desde la perspectiva del equipo multidisciplinario?». La pregunta se estructuró basándose en el acrónimo PCC: Población, Concepto y Contexto: P: población (equipo multidisciplinario); C: concepto (la cultura y el clima organizacional para la SP); C: contexto (servicios de diálisis).

### Criterios de Selección

Se incluyeron estudios que evaluaron la cultura y el clima organizacional para SP mediante instrumentos de medición en servicios de diálisis desde la perspectiva del equipo multidisciplinario, disponibles en su totalidad, de cualquier área geográfica, en bases de datos y en la literatura gris. No hubo restricciones temporales ni de idioma.

Se excluyeron estudios de revisión, investigaciones metodológicas, comentarios, informes institucionales, resúmenes de eventos, manuales técnicos, cartas al editor, informes de casos y reseñas. Estos tipos de documentos no presentaban datos empíricos primarios ni resultados de evaluación necesarios para identificar, describir o analizar la cultura y el clima organizacional. Por lo tanto, se consideraron inadecuados para responder a la pregunta de investigación propuesta en esta revisión de alcance.

### Fuentes de datos y Estrategias de Búsqueda

Las estrategias de búsqueda se diseñaron con la ayuda de un bibliotecario profesional. La búsqueda se realizó el 20 de mayo de 2024 en las bases de datos y repositorios institucionales Literatura Latinoamericana y del Caribe en Ciencias de la Salud (LILACS), Base de Datos de Enfermería (BDENF), Índice Bibliográfico Español de Ciencias de la Salud (IBECS) y ColeccionASUS a través de la Biblioteca Virtual en Salud (BVS), *Medline* a través de *PubMed*, *Cochrane Library, Scopus, Web of Science* y *Embase*. Se accedió a las bases de datos a través del Portal de Revistas de CAPES, mediante la Comunidad Académica Federada (CAFe). También se realizó una búsqueda en la literatura gris en *Google Scholar*, en el Catálogo de Tesis y Disertaciones de la Coordinación de Perfeccionamiento del Personal de Nivel Superior (CAPES) y en los Repositorios de las Universidades (UFC, UFAL, UNB, USP y UFMG). Estos repositorios institucionales fueron seleccionados por la calidad y relevancia de su contenido, así como por el importante papel de las instituciones en la producción y difusión del conocimiento, contribuyendo al avance de la ciencia abierta y a la promoción de investigaciones innovadoras. Las estrategias de búsqueda se adaptaron según las necesidades de las bases de datos consultadas.

Para la selección de los descriptores se utilizaron DeCS (Descriptores en Ciencias de la Salud) /MeSH (*Medical Subject Headings*), con excepción de *Embase*, para la cual se utilizó Emtree. Todos los descriptores incluidos en la estrategia de búsqueda fueron términos controlados, es decir, extraídos de DeCS/MeSH o Emtree. Los operadores booleanos utilizados fueron OR y AND. El operador OR se aplicó para encontrar registros que contuvieran uno de los términos separados (por ejemplo, *hemodialysis or dialysis*) y el AND para encontrar registros que contuvieran todos los términos especificados (por ejemplo, *hemodialysis and ‘patient safety*’), tal y como se muestra en la Cuadro 1.

**Cuadro 1:** Términos y estrategias de búsqueda aplicados a cada base de datos – Belo Horizonte, MG, Brasil, 2024.

Bases de datos y fecha de búsqueda	Estrategias de búsqueda	Número de estudios recuperados
LILACS via BVS 20 de mayo de 2024	(“*Renal Dialysis*” OR “*Diálisis Renal*” OR “Diálise Renal” OR “Diálise Extracorpórea” OR “Hemodiálise” OR “*Dialysis*” OR “*Diálisis*” OR “Diálise” OR “*Hemodialysis Units Hospital*” OR “*Unidades de Hemodiálisis en Hospital*” OR “Unidades Hospitalares de Hemodiálise” OR “Unidades Hospitalares de Diálise Renal” AND (“*Patient Safety*” OR “*Seguridad del Paciente*” OR “Segurança do Paciente” OR “*Organizational Culture*” OR “Cultura Organizacional” OR “Cultura Organizacional” OR “Cultura Corporativa” OR “*Safety Management*” OR “*Administración de la Seguridad*” OR “Gestão da Segurança”) AND (db:(“LILACS” OR “IBECS” OR “BDENF” OR “colecionaSUS”) AND la:(“en” OR “es” OR “pt”))	96
MEDLINE via PUBMED 20 de mayo de 2024	(“*Renal Dialysis*” OR “*Dialysis*” OR “*Hemodialysis Units Hospital*”) AND (“*Patient Safety*” OR “*Organizational Culture*” OR “*Safety Management*”)	597
*Cochrane Library* 20 de mayo de 2024	(“*Renal Dialysis*” OR “*Dialysis*” OR “*Hemodialysis Units Hospital*”) AND (“*Patient Safety*” OR “*Organizational Culture*” OR “*Safety Management*”)	102
*Scopus* 20 de mayo de 2024	(“*Renal Dialysis*” OR “*Dialysis*” OR “*Hemodialysis Units Hospital*”) AND (“*Patient Safety*” OR “*Organizational Culture*” OR “*Safety Management*”)	1745
*Web of Science* 20 de mayo de 2024	(“*Renal Dialysis*” OR “*Dialysis*” OR “*Hemodialysis Units Hospital*”) AND (“*Patient Safety*” OR “*Organizational Culture*” OR “*Safety Management*”)	389
*Embase* 20 de mayo de 2024	*hemodialysis* or *dialysis* AND *‘patient safety’*	2708
**Literatura gris y fecha de búsqueda**	**Estratégias de búsqueda**	**Número de estudios recuperados**
*Google Scholar* 20 de mayo de 2024	(Percepção) AND (“Cultura de segurança do paciente” OR “Clima de segurança do paciente”) AND (“Serviços de diálise”)	8000
Catálogo de teses e dissertações da CAPES 20 de mayo de 2024	(Percepção) AND (“Cultura de segurança do paciente” OR “Clima de segurança do paciente”) AND (“Serviços de diálise”)	0
Repositorios (UFC, UFAL, UNB, USP, UFMG) 20 de mayo de 2024	(Percepção) AND (“Cultura de segurança do paciente” OR “Clima de segurança do paciente”) AND (“Serviços de diálise”)	66

### Selección de los Estudios

Dos investigadores seleccionaron los estudios de forma independiente y un tercero evaluó las divergencias hasta alcanzar un consenso final. Se utilizó el gestor de referencias *Endnote*, donde se excluyeron los duplicados. A partir de ahí, la lista de referencias se exportó al gestor bibliográfico Rayyan Qatar Computing Research Institute (Rayyan QCRI); el proceso de selección de los estudios se llevó a cabo en tres etapas: análisis de los títulos, lectura de los resúmenes y lectura de los estudios completos. Estas etapas se guiaron por los criterios de inclusión y exclusión. El mismo proceso se llevó a cabo para la selección de los artículos de la literatura gris, y se analizó la búsqueda manual en las listas de referencias de los estudios incluidos como estrategia para identificar producciones científicas que no se habían recuperado anteriormente.

### Extracción y Análisis de datos

Los investigadores elaboraron una base de datos con el programa *Microsoft Excel®* versión 2016 para extraer los siguientes datos: autor/año de publicación; título; país donde se realizó el estudio; objetivos; muestra; tasa de adhesión de los participantes; población analizada; lugar del estudio; instrumento utilizado para evaluar la cultura o el clima organizacional para la SP; nivel de cultura o clima de SP; fortalezas; debilidades y oportunidades de mejora para la cultura y el clima organizacional para la SP en los servicios de diálisis.

Los datos extraídos y las principales características de los artículos seleccionados se recopilaron en la Cuadro 2. Para analizar y categorizar los resultados de los estudios que utilizaron el HSOPSC^(23)^ y el MOPSC^(22)^ y sus versiones adaptadas, se utilizaron los criterios de evaluación propuestos por la *Agency for Healthcare Research and Quality* (AHRQ)^(23)^: las áreas consideradas fortalezas o fuertes son aquellas que tienen un 75% o más de respuestas positivas (PRP) del equipo multidisciplinario, y las áreas consideradas frágiles o con debilidades son aquellas en las que el PRP es igual o inferior al 50%. Las áreas evaluadas con <75% y >50% se consideran oportunidades de mejora para la cultura y el clima organizacional de la SP^(23)^. En cuanto al análisis de los instrumentos SAQ versión adaptada^(30)^ y *Safety Climate Survey* (SCS)^(31)^, los autores consideraron áreas fuertes o con fortalezas aquellas con un PRP del 75% o más, y áreas frágiles o con debilidades aquellas con un PRP inferior al 75%^(30,31)^.

**Cuadro 2:** Caracterización de los estudios incluidos en la revisión del alcance (n = 12) – Belo Horizonte, MG, Brasil, 2024.

Autor, año y país	Muestra y tasa de adhesión	Objetivos de los estudios	Población	Local del servicio
Hoffmann et al.^(32)^ (2023); Brasil	n = 56 (%) = 71,8	Evaluar la cultura de SP desde la perspectiva del equipo de enfermería en servicios de diálisis durante la pandemia de COVID-19.	Enfermeros, técnicos y auxiliares de enfermería.	Tres servicios de diálisis de Minas Gerais
Rodrigues et al.^(24)^ (2023); Brasil	n = 134 (%) = 86	Evaluar los factores asociados a la cultura de SP en diálisis durante la pandemia de COVID-19.	Enfermeros, técnicos y auxiliares de enfermería, asistentes sociales, nutricionistas, psicólogos, farmacéuticos y el sector administrativo.	Tres servicios de diálisis de Minas Gerais
Silva^(33)^ (2019); Brasil	n = 32 (%) = 89	Evaluar la cultura de SP de los profesionales en un servicio de hemodiálisis de un hospital universitario.	Enfermeros, técnicos y auxiliares de enfermería, médicos, personal administrativo, nutricionistas, personal de limpieza y higiene, psicólogos, asistentes sociales, recepcionistas y técnicos de agua.	Hospital Universitario de Bahía
Millson; Hackbarth; Benard^(34)^ (2019); EE. UU	n = 99 (%) ≥80	Evaluar las percepciones del equipo de hemodiálisis sobre la cultura de SP, implementar el programa de seguridad de la AHRQ y promover una mayor adhesión del equipo a las normas de prevención de infecciones.	Enfermeros, técnicos y auxiliares de enfermería.	Seis clínicas de diálisis ambulatoria de Nueva York
Grilo^(35)^ (2018); Portugal	n = 108 (%) = 73	Analizar la percepción de los profesionales de la salud sobre la cultura de SP; comparar las percepciones entre las diferentes categorías profesionales; e identificar áreas para mejorar la cultura de SP.	Enfermeros, técnicos y auxiliares de enfermería, médicos y administradores	Clínica de hemodiálisis en la región de Lisboa
Izquierdo et al.^(36)^ (2018); España	n = 111 (%) = 85	Determinar la frecuencia de las percepciones y actitudes de los profesionales de los centros de hemodiálisis en relación con la SP; e identificar puntos fuertes y oportunidades de mejora para la SP.	Enfermeros, auxiliares de enfermería y otras categorías	Seis centros de hemodiálisis de Castilla-La Mancha
Dantas^(37)^ (2018); Brasil	n = 38 (%) = 100	Evaluar la cultura de SP en unidades de hemodiálisis desde la perspectiva del equipo de enfermería; y cuantificar los eventos reportados por este equipo.	Enfermeros, técnicos y auxiliares de enfermería	Dos unidades hospitalarias de hemodiálisis en Ceará
Davis et al.^(38)^ (2016); EE. UU	n = 581 (%) = 97	Evaluar las percepciones del equipo multidisciplinario sobre la cultura de SP en clínicas de hemodiálisis.	Sector administrativo, médicos, nutricionista, asistente social, enfermeros y técnicos de enfermería.	Clínicas de hemodiálisis en dos ciudades de EE. UU
Ulrich; Kear^(39)^ (2015); EE. UU	n = 929 (%) = 95	Analizar las percepciones de los enfermeros nefrológicos sobre SP y su cultura, considerando la unidad de trabajo, el ambiente organizacional y el papel desempeñado.	Enfermeros	Asociación Americana de Enfermeras de Nefrología
Ulrich; Kear^(40)^ (2014); EE. UU	n = 929 (%) = 95	Describir los resultados del cultivo de SP en entornos de práctica de enfermería nefrológica; y comparar los datos del cultivo de SP obtenidos con los datos publicados por la AHRQ.	Enfermeros	Asociación Americana de Enfermeras de Nefrología
Taher et al.^(41)^ (2014); Arabia Saudita	n = 509 (%) = 65	Evaluar el clima de SP percibido por enfermeros y médicos en unidades de diálisis en Arabia Saudita.	Enfermeros y médicos	Cinco unidades de diálisis en tres ciudades de Arabia Saudita
Di Benedetto et al.^(42)^ (2011); Italia	n = 346 (%) = 89	Evaluar el nivel del clima de SP en la red de clínicas de diálisis y la adhesión del equipo de salud a la ejecución de los procedimientos estándar para mejorar la seguridad en el servicio de diálisis.	Enfermeros, auxiliares de cuidados y médicos	Treinta y tres centros de diálisis de *Fresenius Medical Care* en Italia

SP: Seguridad del Paciente; COVID-19: *Coronavirus Disease* 2019; AHRQ: *Agency for Healthcare Research and Quality*; EE. UU.: Estados Unidos de América.

Los estudios seleccionados se organizaron por año de publicación, y las áreas de fortalezas, oportunidades de mejora y debilidades se presentaron junto con el instrumento de medición utilizado en formato de cuadro.

Todos los investigadores participaron en la interpretación y síntesis de los datos de los artículos, utilizando la discusión narrativa para asociar los resultados tabulados con el objetivo y la pregunta de investigación orientadora.

## RESULTADOS

En las bases de datos consultadas se encontraron un total de 5637 estudios. Tras eliminar 2476 duplicados, quedaron 3161 estudios. De estos, 3123 fueron excluidos tras leer los títulos y los resúmenes. No se seleccionó ninguna evidencia tras la búsqueda manual en la lista de referencias de los estudios incluidos. De los 38 estudios seleccionados para su lectura completa, se excluyeron 29 mediante justificaciones, lo que dio lugar a la inclusión de nueve estudios. En la literatura gris se identificaron 8066 estudios. Tras la lectura de los títulos y resúmenes, se excluyeron 7792 estudios por no responder a la pregunta orientadora y a los criterios de inclusión de esta revisión de alcance. Así, se evaluaron 274 para su elegibilidad, de los cuales se excluyeron 271 mediante justificaciones, lo que dio como resultado la inclusión de tres estudios en la muestra. Finalmente, se encontraron 13703 estudios, se leyeron 312 en su totalidad y se seleccionaron 12 para componer la muestra final. Todo este proceso se describe en el diagrama de flujo PRISMA ScR (Figura 1).

**Figura 1:** Flujograma PRISMA-ScR de selección de estudios para su inclusión en la revisión del alcance – Belo Horizonte, MG, Brasil, 2024.

Los 12 estudios^(24,32–42)^ incluidos en esta revisión del alcance presentaron un enfoque metodológico cuantitativo y un diseño transversal, cuyos objetivos se detallan en el Cuadro 2. El periodo de publicación fue entre 2011 y 2023 y todos se desarrollaron en servicios de diálisis públicos, privados o filantrópicos. La mayoría de las investigaciones eran de origen estadounidense y brasileño (cuatro estudios de cada uno, lo que corresponde al 33,4% del total en ambos casos). La tasa de adhesión de los participantes a los estudios varió entre el 65% y el 100%, con una media del 85,5%. En el ámbito del equipo multidisciplinario, la composición de los participantes difirió entre los estudios analizados; dos estudios estadounidenses^(39,40)^ evaluaron exclusivamente la percepción de los enfermeros de nefrología (Cuadro 2).

En cuanto a los instrumentos de evaluación, la mayoría (n = 9; 75%) utilizó instrumentos para evaluar la cultura organizacional para SP, entre los cuales el HSOSPC^(23)^ fue el más referenciado^(24,32–36,38–40)^. Los demás (n = 3; 25%) emplearon instrumentos para medir el clima organizacional para SP, destacándose el SAQ^(37)^ y el SCS^(41,42)^.

Las principales fortalezas identificadas se relacionaron con el trabajo en equipo en los servicios de diálisis^(35,36,38–40)^. A continuación, se destacaron el aprendizaje organizacional y la mejora continua (capacitaciones), así como el Retroalimentación y la comunicación sobre errores, presentes en el 75% de los estudios (n = 9)^(24,33–36,38–41)^. Tres estudios internacionales (25%)^(38–40)^ señalaron la importancia de las acciones para el control de infecciones en las prácticas nefrológicas como áreas fortalecidas para la cultura y el clima organizacional para la SP en los servicios de diálisis (Cuadro 3).

**Cuadro 3:** Dimensiones de la cultura y el clima organizacional para la seguridad del paciente (n = 12) – Belo Horizonte, MG, Brasil, 2024.

Artículos	Instrumento de evaluación de la cultura y del clima organizacional para SP	Categorías	Dimensiones de los instrumentos de evaluación de la cultura y el clima organizacional para SP
Hoffman et al.^(32)^ (2023); Brasil	HSOSPC adaptado	Fortalezas	Expectativas y acciones de promoción de la seguridad por parte de la dirección/supervisión/gerentes de la unidad/servicio (78%)
		Oportunidades de mejorar	Aprendizaje organizacional/mejora continua (69%) Retroalimentación y comunicación sobre errores (63%) Trabajo en equipo dentro de las unidades/servicios (63%) Comunicación abierta (55%)
		Fragilidades	Traslados internos y cambios de turno (49%) Dotación de personal (45%) Percepción general de la SP (45%) Apoyo de la dirección del hospital a la SP (48%) Trabajo en equipo entre las unidades (45%) Frecuencia de eventos adversos notificados (43%) Respuestas no punitivas a los errores (22%)
Rodrigues et al.^(24)^ (2023); Brasil	HSOSPC adaptado	Fortalezas	Aprendizaje organizacional/mejora continua (86%) Expectativas y acciones de promoción de la seguridad por parte de la dirección/supervisión/gerentes de la unidad/servicio (77%)
		Oportunidades de mejorar	Trabajo en equipo dentro de las unidades/servicios (73%) Apoyo de la dirección del hospital a la SP (64%) Retroalimentación y comunicaciones sobre errores (60%) Frecuencia de eventos adversos notificados (59%) Traslados internos y cambios de turno (57%) Comunicación abierta (56%) Trabajo en equipo entre las unidades (56%) Percepción general de la SP (54%)
		Fragilidades	Dotación de personal (49%) Respuestas no punitivas a los errores (29%)
Silva^(33)^ (2019); Brasil	HSOSPC adaptado	Fortalezas	Aprendizaje organizacional y mejora continua (84%)
		Oportunidades de mejorar	Trabajo en equipo dentro de las unidades/servicios (74%) Dotación de personal (58%)
		Fragilidades	Respuestas no punitivas a los errores (47%) Retroalimentación y comunicación sobre los errores (46%) Apoyo de la dirección del hospital a la SP (45%) Frecuencia de los eventos adversos notificados (44%) Comunicación abierta (38%) Expectativas y acciones de promoción de la seguridad por parte de la dirección/supervisión/gerentes de la unidad/servicio (35%) Trabajo en equipo entre las unidades (32%) Percepción general de la SP (26%) Transferencias internas y cambios de turno (19%)
Millson; Hackbart; Benard (2019)^(34)^; EE. UU	HSOSPC adaptado	Fortalezas	Aprendizaje organizacional y mejora continua (80%) Apertura de la comunicación (77%)
		Oportunidades de mejorar	Apoyo de la dirección hospitalaria a la SP (63%) Trabajo en equipo entre las unidades (57%)
		Fragilidades	No mencionadas
Grilo^(35)^ (2018); Portugal	HSOSPC adaptado	Fortalezas	Trabajo en equipo dentro de las unidades (81%) Aprendizaje organizacional y mejora continua (77%) Apoyo de la dirección hospitalaria a la SP (75%) Trabajo en equipo entre las unidades (75%)
		Oportunidades de mejorar	Percepción general de SP (72%) Expectativas y acciones de promoción de la seguridad por parte de la dirección/supervisión/gerentes de la unidad/servicio (67%) Traslados internos y cambios de turno (61%) Dotación de personal (55%) Frecuencia de eventos adversos notificados (58%)
		Fragilidades	Retroalimentación *y* comunicación sobre errores (50%) Comunicación abierta (50%) Respuestas no punitivas a los errores (37%)
Izquierdo et al.^(36)^ (2018); España	HSOSPC adaptado	Fortalezas	Trabajo en equipo dentro de las unidades (86%) Retroalimentación *y* comunicación sobre errores (76%) Expectativas y acciones de promoción de la seguridad por parte de la dirección/supervisión/gerentes de la unidad/servicio (75%)
		Oportunidades de mejorar	Frecuencia de eventos adversos notificados (Dimensionamento de pessoal73%) Aprendizaje organizacional y mejora continua (71%) Percepción general de la SP (70%) Comunicación abierta (65%) Dotación de personal (63%) Respuestas no punitivas a los errores (63%) Traslados internos y cambios de turno (62%) Apoyo de la dirección del hospital a la SP (59%) Trabajo en equipo entre las unidades (55%)
		Fragilidades	No mencionadas
Dantas^(37)^ (2018); Brasil	SAQ adaptado	Fortalezas	Satisfacción en el trabajo (86%) Ambiente de trabajo en equipo (82%) Condiciones de trabajo (79%)
		Oportunidades de mejorar	No mencionadas
		Fragilidades	Clima de SP (72%) Percepción de estrés (67%)
Davis et al.^(38)^ (2016); EE. UU	HSOSPC adaptado	Fortalezas	Apoyo de la dirección del hospital a la SP (88%) Cuando hay mucho que hacer rápidamente, somos capaces de trabajar en equipo (86%) Los directivos tienen en cuenta las sugerencias de los trabajadores sobre las medidas para prevenir las infecciones asociadas al acceso vascular (84%) Se discuten formas de evitar que se repitan los errores en la unidad (83%) Estamos modificando activamente los protocolos y políticas para reducir las infecciones relacionadas con el acceso vascular en nuestra institución (82%) Después de realizar cambios para mejorar la SP, evaluamos su eficacia (81%) Mi supervisor o gerente ofrece retroalimentación positiva cuando identifica que el trabajo se ha realizado de acuerdo con los procedimientos establecidos para la SP (79%) Los trabajadores se sienten cómodos para hablar si observan algo que pueda aumentar el riesgo de infecciones relacionadas con el acceso vascular (76%) Los errores pueden conducir a cambios positivos (76%) Tratarse mutuamente con respeto (75%)
		Oportunidades de mejorar	No mencionadas
		Fragilidades	No mencionadas
Ulrich; Kear^(39)^ (2015); EE. UU	HSOSPC e MOSPC adaptados	Fortalezas	Trabajo en equipo (87%) Formación del equipo (80%) Control de infecciones en diálisis (76%) Liderazgo continuo: aprendizaje organizacional (76%)
		Oportunidades de mejorar	Apoyo de la dirección del hospital a la SP (74%) Expectativas y acciones de promoción de la seguridad por parte de la dirección/supervisión/gerentes de la unidad/servicio (70%) Estandarización de procesos (69%) Percepción general de la SP (65%) Comunicación abierta (64%) Retroalimentación y comunicación sobre errores (63%) Apoyo organizacional de la dirección a la SP (60%) Frecuencia de los eventos adversos notificados (55%) Respuestas no punitivas a los errores (55%) Dotación de personal (54%)
		Fragilidades	No mencionadas
Ulrich; Kear^(40)^ (2014); EE. UU	HSOSPC adaptado	Fortalezas	Trabajo en equipo entre las unidades (81%) Control de infecciones (79%) Formación del equipo (78%)
		Oportunidades de mejorar	Aprendizaje organizacional y mejora continua (73%) Expectativas y acciones de promoción de la seguridad por parte de la dirección/supervisión/gerentes de la unidad/servicio (72%) Apoyo de la dirección del hospital a la SP (67%) Percepción general de la SP (65%) Estandarización de procesos (62%) Comunicación abierta (60%) Retroalimentación y comunicación sobre errores (59%) Dotación de personal (57%) Apoyo organizacional de la dirección a la SP (56%) Respuestas no punitivas a los errores (55%) Frecuencia de los eventos adversos notificados (55%)
		Fragilidades	Transferencias internas y traslados de guardia (33%)
Taher et al.^(41)^ (2014); Arabia Saudita	SCS	Fortalezas	El traspaso de información al inicio del turno es importante para la SP (85%) El personal asume la responsabilidad de la SP (84%) La SP se considera una prioridad (83%) La comunicación de información es frecuente en este entorno (81%) Conozco bien los canales para resolver dudas sobre cuestiones relacionadas con la SP (79%) Los eventos adversos suelen estar asociados a múltiples fallos en el sistema (78%) Los médicos y enfermeros líderes de mi área clínica me escuchan y tienen en cuenta mis preocupaciones (77%) Mis compañeros me animan a comunicar cualquier preocupación que pueda tener sobre la SP (76%) Se han realizado más esfuerzos en materia de SP en comparación con el año pasado (76%)
		Oportunidadesde mejorar	No mencionadas
		Fragilidades	El liderazgo nos está llevando a actuar como una institución centrada en la SP (74%) Los errores médicos se tratan adecuadamente (69%) La cultura de SP de esta área clínica propicia el aprendizaje basado en los errores (68%) Me sentiría seguro al ser tratado en esta unidad (67%) Recibo una retroalimentación adecuada sobre mi desempeño (64%) La dirección no ignora las cuestiones de SP en aras de la productividad (62%) Mis sugerencias sobre SP son tenidas en cuenta por la dirección (68%)
Di Benedetto et al.^(42)^ (2011) – Itália	SCS	Fortalezas	El liderazgo nos está llevando a actuar como una institución centrada en la SP (93%) Los médicos y enfermeros líderes de mi área clínica me escuchan y tienen en cuenta mis preocupaciones (83%) Mis compañeros me animan a comunicar cualquier preocupación que pueda tener sobre la SP (85%) La cultura de SP de esta área clínica fomenta el aprendizaje basado en los errores (82%)
		Oportunidades de mejorar	No mencionadas
		Fragilidades	No mencionadas

SP: Seguridad del Paciente; HSOPSC: *Hospital Survey on Patient Safety Culture*; SAQ: *Safety Attitudes Questionnaire*; MOSPSC: *Medical Office Survey on Patient Safety Culture*; SCS: *Safety Climate Scale*.

El tipo de gestión de los servicios también se evaluó como un factor que influye en la percepción de los profesionales. Dos estudios (17%) brasileños^(24,32)^ identificaron los factores laborales que predisponen a la cultura y al clima organizacional para la SP en las prácticas asistenciales nefrológicas. Uno de los estudios^(24)^ identificó más áreas fuertes en los servicios de diálisis con gestión privada y filantrópica; las áreas frágiles, por su parte, se reportaron con mayor frecuencia en el servicio de gestión pública, según la percepción del equipo multidisciplinario. Sin embargo, otro estudio^(32)^ señaló que el equipo de enfermería del servicio de diálisis público presentaba una mejor percepción de la cultura y el clima organizacional para la SP en comparación con los equipos de los servicios de diálisis filantrópicos y privados.

En la mayoría de los estudios (n = 8; 67%), la percepción general de la cultura y el clima organizacional para la SP fue evaluada por los participantes como positiva^(24,32,35,38–42)^. Sin embargo, dos estudios brasileños (n = 2; 17%) evaluaron la percepción general como predominantemente negativa^(33,37)^.

## DISCUSIÓN

A partir de los resultados, se identificó que los estudios más recientes sobre la evaluación de la cultura y el clima organizacional para la SP en los servicios de diálisis se publicaron entre los años 2019 y 2023, de los cuales tres eran brasileños^(24,32,33)^ y uno estadounidense^(34)^. Cabe destacar que dos estudios se desarrollaron durante la pandemia de *Coronavirus Disease* 2019 (COVID-19) en Brasil, uno centrado en la percepción del personal de enfermería^(32)^ y otro en la del equipo multidisciplinario^(24)^. Así, ante las innumerables dificultades experimentadas en el contexto pandémico para la producción científica en esta área, es imprescindible que los gestores y formuladores de políticas públicas promuevan la construcción y la implementación de estrategias para mejorar la cultura y el clima organizacional para la SP en los entornos de prácticas nefrológicas^(16)^.

La mayoría de los estudios se realizaron en países desarrollados, en los que la cultura y el clima organizacional para la SP se evaluaron mejor en comparación con los estudios realizados en países de ingresos bajos o medios^(38,42)^. Brasil fue el único país de América Latina donde se pudieron encontrar estudios sobre el tema. Ante este panorama, se plantea el debate sobre la interferencia negativa de las desigualdades socioeconómicas y geográficas en la percepción de la cultura y el clima organizacional para la SP por parte del equipo multidisciplinario de los servicios de diálisis. De manera similar, los estudios han demostrado que las diversas desigualdades interfieren negativamente en el acceso a los servicios de salud y en la calidad de la asistencia prestada, lo que constituye un problema de salud pública^(43,44)^.

Evaluar la cultura y el clima organizacional para la SP debe ser una prioridad de los gestores y profesionales de los servicios de salud, ya que esto permite identificar y medir las condiciones organizacionales que pueden propiciar el aumento de la AE e impactar en la calidad de la atención ofrecida, además de estimular el desarrollo de estrategias de mejora de la SP en la Red de Atención en Salud^(45)^. Los instrumentos de evaluación de la cultura y el clima de la SP pueden orientar la comprensión y la transformación de las actitudes y el comportamiento relacionados con la SP, a partir de la percepción de los profesionales de una organización de salud^(45)^.

Las áreas más destacadas de la cultura y el clima organizacional para la SP fueron: el aprendizaje organizacional y la mejora continua; la retroalimentación y la comunicación sobre los errores; y el trabajo en equipo. Una cultura y un clima organizacional positivos para la SP pueden orientar el comportamiento del equipo hacia la prestación de una atención segura. Por lo tanto, las áreas fuertes destacadas en los estudios deben ser prioritarias en los servicios de salud. De este modo, se contribuye a animar a los profesionales a realizar análisis de errores y AE, con el objetivo de mejorar continuamente la SP en los servicios de salud^(46)^.

Específicamente en los servicios de diálisis, los profesionales reconocen que el fortalecimiento de la cultura y el clima organizacional para la SP propicia la mejora y el desarrollo de competencias esenciales para una práctica asistencial segura^(24)^. En esta perspectiva, los servicios de salud deben sustituir la cultura punitiva del error por una cultura de aprendizaje, que fomente la comunicación abierta de los incidentes sin represión ni castigo^(47)^.

La fuerza del trabajo en equipo conecta los conocimientos y habilidades de diferentes personas con un único fin: la asistencia al paciente^(45)^. Sin embargo, el trabajo del equipo multidisciplinario en salud presenta desafíos como la existencia de conflictos, el individualismo y la jerarquización de las relaciones^(48)^. Es importante que los profesionales adopten medidas para el desarrollo adecuado del trabajo en equipo, con enfoque en la contribución a la calidad y el acceso a los servicios de salud^(49)^.

Estudios internacionales^(38–40)^ han demostrado la prevención de infecciones en los servicios de diálisis para promover la mejora de la cultura y el clima organizacional para la SP; por ello, el riesgo de infección se considera una de las principales causas de mortalidad y hospitalización en pacientes en tratamiento dialítico^(5)^. Se necesitan estrategias como la implementación del análisis investigativo (causa raíz) para determinar los problemas de los sistemas y procesos a fin de reducir y mitigar los riesgos y los EA en los servicios de diálisis^(16)^. Además, se deben priorizar acciones específicas en el área de la higiene de las manos y el cuidado de los catéteres venosos centrales para el control efectivo de las infecciones^(16,26)^.

Estudios brasileños^(24,32,33,37)^ presentaron áreas relacionadas con la percepción general de la cultura y el clima organizacional para la SP como una fragilidad u oportunidad de mejora. Esto puede estar asociado a los retos profesionales de los servicios públicos brasileños, como la alta demanda de atención y la falta de mayores inversiones en recursos humanos y tecnológicos^(50)^.

Tres estudios (25%) evidenciaron la dotación de personal y la transición/transferencia/cambio de turno entre unidades y servicios de diálisis como debilidades^(24,32,40)^. Sin embargo, cuatro estudios (33%) identificaron estas áreas como oportunidades de mejora para la cultura y el clima organizacional para la SP^(33,35,36,39)^. En dos estudios (17%), las condiciones de trabajo se consideraron áreas fuertes^(37,41)^. Cabe destacar que la dotación de personal en los servicios de diálisis en Brasil está prevista en la Resolución de la Dirección Colegiada (RDC) n.º 11/2014, que también establece los requisitos de buenas prácticas de funcionamiento para los servicios de diálisis^(51)^.

Un estudio brasileño con el equipo de enfermería puso de manifiesto que las condiciones de trabajo inadecuadas, derivadas del entorno y de las funciones desempeñadas, repercuten negativamente en la percepción de la cultura y el clima organizacional en SP^(52)^. El ambiente de trabajo genera desgaste físico y mental debido a la falta de reconocimiento profesional, la falta de motivación, la falta de insumos, la remuneración inadecuada, la carga horaria excesiva, la dotación incorrecta de profesionales, el trabajo nocturno y la insatisfacción de los profesionales^(52)^.

Cabe destacar que el cambio de turno es el momento en que el equipo multidisciplinario discute la información más importante relacionada con la evolución de los pacientes, lo que puede contribuir a la continuidad de la atención y a garantizar la SP, siempre que se desarrolle mediante una comunicación eficaz^(53)^. Para ello, es imprescindible la estandarización mediante protocolos y herramientas de comunicación que garanticen la continuidad de las acciones, además de incentivar los cambios de actitud y fortalecer la cultura y el clima organizacional^(52)^.

El apoyo de los gestores y los líderes a las preocupaciones, demandas y sugerencias del equipo multidisciplinario que trabaja en los servicios de diálisis se destacó como una dimensión frágil o como una oportunidad de mejora en el 75% (n = 9) de los estudios^(24,32–34,36,39–42)^. La participación del liderazgo en la acogida de las demandas y sugerencias del equipo multidisciplinario es de suma importancia en la promoción de la SP. Un estudio iraní realizado en hospitales señala que los gerentes, líderes y supervisores deben promover acciones en favor del desarrollo de valores, creencias y comportamientos personales alineados con los valores de la cultura y el clima organizacional. El compromiso del líder en la identificación de problemas en el proceso de trabajo y en el desarrollo de estrategias que subsidien recursos y oportunidades de aprendizaje puede contribuir a la mejora de la prestación de los cuidados de salud^(46)^.

Además, el modelo de gestión adoptado por la organización de salud puede influir en la percepción de los profesionales en relación con la cultura y el clima organizacional para la SP. En este sentido, es necesario que todos estén comprometidos con el mantenimiento y la promoción de la cultura y el clima de la SP. Por lo tanto, la gestión tiene un papel esencial en la mejora de la cultura y el clima organizacional para la SP, en la medida en que promueve la organización de flujos que mejoran la eficacia del trabajo en salud^(54)^.

Las dimensiones de la cultura y el clima organizacional para la SP «frecuencia de EA notificados» y «respuestas no punitivas a los errores» fueron citadas como oportunidad de mejora o fragilidad en siete estudios (58%)^(24,32,33,35,36,39,40)^. La dimensión «los EA se asocian frecuentemente con múltiples fallos en el sistema» se consideró una fortaleza en tres (25%) investigaciones^(36,38,41)^. Así, se evidencia que la existencia de una cultura punitiva y la sobrecarga de trabajo inhiben a los profesionales a la hora de notificar los EA. Estos resultados concuerdan con otro estudio, en el que la cultura punitiva y la carga de trabajo excesiva también se consideran obstáculos para la notificación de EA^(53)^. Desde esta perspectiva, la subnotificación de EA debe abordarse como una oportunidad de mejora, ya que impide el desarrollo de acciones para evitar la recurrencia de errores en salud y el diagnóstico de riesgos del servicio.

La influencia de la categoría profesional en la percepción de la cultura y el clima organizacional para la SP fue analizada por estudios con resultados diferenciados. En cinco artículos (42%), los administradores/gestores/líderes presentaron una percepción general más positiva de la cultura y el clima organizacional para la SP en comparación con el resto del equipo multidisciplinario de diálisis^(24,35,38,40,42)^. Dos estudios (17%) identificaron que los médicos, el equipo multidisciplinario y el sector administrativo informaron más áreas de fortalezas en comparación con el equipo de enfermería^(24,38)^. Dos estudios brasileños destacaron que los enfermeros, en comparación con los médicos o auxiliares y técnicos de enfermería, presentaron una mejor evaluación de la percepción de la cultura y el clima organizacional para la SP^(32,37).^ Un estudio realizado en Arabia Saudita también corrobora este hallazgo^(41)^. Por el contrario, un estudio brasileño^(33)^ señala que la percepción entre los auxiliares y técnicos de enfermería fue mejor evaluada en comparación con la de los enfermeros y médicos de los servicios de diálisis. Dos estudios estadounidenses evaluaron la cultura y el clima organizacional para la SP desde la perspectiva de 929 enfermeros que trabajaban en servicios de diálisis^(39,40)^. La percepción de los enfermeros se considera esencial, dada su proximidad con los pacientes, su función de liderazgo y su papel central en el control de infecciones y la comunicación eficaz en el equipo^(24,39,40)^.

Por lo tanto, en los estudios se observó la influencia de la categoría profesional en la percepción de la cultura y el clima organizacional para la SP en los servicios de diálisis. Sin embargo, no fue posible identificar un patrón de cómo se producen estas influencias a partir de los estudios mapeados. Un estudio internacional identificó que los gerentes y el sector administrativo presentaban una percepción más positiva de la cultura y el clima de la SP en comparación con los demás miembros del equipo multidisciplinario^(55)^.

En este sentido, se observó una variabilidad en la percepción del equipo multidisciplinario en el contexto brasileño. Un estudio identificó una percepción más positiva sobre la cultura y el clima organizacional para la SP en los servicios de diálisis por parte de los gerentes y el sector administrativo^(24)^. Dos estudios destacaron la percepción de los enfermeros en comparación con la de los médicos y otros miembros del equipo de enfermería^(32,37)^. Por último, uno identificó una mejor percepción entre los técnicos y auxiliares de enfermería en comparación con los enfermeros y médicos^(33)^. Sin embargo, las desigualdades en la percepción de los profesionales sobre la cultura y el clima organizacional para la SP deben corregirse mediante su valoración profesional y debates sobre prácticas que fomenten la atención segura^(56)^.

Además, es necesario que el tema se aborde en las directrices curriculares nacionales y en el plan político pedagógico de la formación profesional en el área de la salud durante el proceso de enseñanza-aprendizaje, ya que es esencial para el fortalecimiento de la cultura y el clima de la SP, dado que los estudiantes actuales serán los futuros profesionales de los servicios de salud^(57)^, incluidos los servicios de diálisis.

La categoría profesional también influyó en la percepción de la cultura y el clima organizacional para la SP según el modelo de gestión de los servicios de diálisis^(24,32)^. Estas diferencias pueden justificarse por algunas hipótesis, ya que las instituciones privadas reciben una mayor inversión de los gestores en acciones de mejora de los procesos de trabajo y de la calidad de la asistencia prestada debido a la competitividad del mercado^(24)^. Por otro lado, el equipo de enfermería desarrolla habilidades y competencias dirigidas a la identificación de riesgos que implican la SP debido a la proximidad con el paciente durante la asistencia prestada, en comparación con otras categorías profesionales^(52)^. Por último, el servicio público de diálisis durante la pandemia recibió capacitación sobre los flujos de atención, ya que se trata de un hospital de referencia para el tratamiento de la COVID-19, lo que puede haber influido en la percepción del personal de enfermería^(32)^.

Ante esto, los resultados de los estudios mapeados evidenciaron que la cultura y el clima organizacional para la SP en los servicios de diálisis son percibidos por el equipo multidisciplinario como relativamente positivos o neutros (oportunidades de mejora). Sin embargo, se espera encontrar una cultura y un clima organizacional para la SP positivos en todos los servicios, ya que su consolidación es fundamental para mejorar la calidad de la atención. Para ello, es necesario que los gestores y líderes reconozcan la importancia de la comunicación para identificar las preocupaciones de los profesionales de la asistencia y de los pacientes^(52)^. Además, la colaboración del equipo multidisciplinario es indispensable para mantener las fortalezas, perfeccionar las oportunidades de mejora y reparar las debilidades en las áreas de la cultura y el clima organizacional para la SP en los servicios de diálisis.

Se ha observado que la literatura sobre este tema es incipiente y debe valorarse en el contexto mundial, ya que se han identificado pocos estudios que evalúen la cultura y el clima organizacional de la SP en los servicios de diálisis desde la perspectiva del equipo multidisciplinario. Cabe destacar como limitación el hecho de que dos estudios incluidos^(39,40)^ abordaron exclusivamente la percepción de los enfermeros, lo que puede restringir la generalización de los hallazgos a todo el equipo multidisciplinario.

Otro punto importante es la falta de consenso en la literatura sobre el uso de los términos «cultura» y «clima organizacional» para la SP, aspecto que dificultó el proceso de análisis de los datos de los estudios de esta revisión. Los investigadores minimizaron la dificultad al considerar ambos términos, ya que están intrínsecamente correlacionados y se reflejan en la práctica asistencial y gerencial de los servicios de diálisis. Sin embargo, esta revisión contribuirá a la práctica clínica y gerencial de los servicios de diálisis en la medida en que promueve una mayor comprensión de la cultura y el clima organizacional para la SP desde la perspectiva del equipo multidisciplinario.

## CONCLUSIÓN

Las mayores fortalezas de la cultura y el clima organizacional para la SP se encontraron en las áreas de trabajo en equipo, aprendizaje organizacional y mejora continua, retroalimentación y comunicación sobre errores. Las debilidades y oportunidades de mejora se referían a la dotación de personal, la transición de turnos y las transferencias internas, el apoyo de los líderes, la notificación de eventos adversos y las respuestas no punitivas a los errores.

La categoría profesional, la ubicación geográfica y el tipo de gestión de los servicios de diálisis influyeron en la percepción de la cultura y el clima organizacional para la SP, y estos fueron positivos en la mayoría de los estudios. Los hallazgos de esta revisión pueden ayudar a los gestores, líderes y profesionales en la planificación estratégica de la atención prestada en los servicios de diálisis, a partir de la evaluación de la cultura y el clima organizacional para la SP, con el fin de proponer acciones de mejora continua de la SP y la atención en salud. De este modo, contribuirá a orientar futuros estudios que promuevan el fortalecimiento de la cultura y el clima de la SP y de las prácticas nefrológicas seguras en el ámbito de la salud pública brasileña. Los hallazgos señalaron áreas de debilidad, pero no presentaron una comprensión detallada de los factores que contribuyen a tales resultados, lo que suscita la necesidad de realizar nuevos estudios con el fin de obtener una mayor caracterización de cómo el equipo multidisciplinario evalúa y percibe la cultura y el clima organizacional para la SP en los servicios de diálisis renal, así como una posible generalización de los resultados.

## DISPONIBILIDAD DE DATOS

Todo el conjunto de datos que respalda los resultados de este estudio se puso a disposición y se publicó en el propio artículo.
